# Risk factors for medication-related short-term readmissions in adults – a scoping review

**DOI:** 10.1186/s12913-023-10028-2

**Published:** 2023-09-28

**Authors:** N. Schönenberger, C. Meyer-Massetti

**Affiliations:** 1 Clinical Pharmacology and Toxicology, Department of General Internal Medicine, Inselspital, Bern University Hospital, University of Bern, Bern, Switzerland; 2 Graduate School for Health Sciences, University of Bern, Bern, Switzerland; 3 Institute of Primary Healthcare (BIHAM), University of Bern, Bern, Switzerland

**Keywords:** Drug-related readmissions, Drug-related problems, Adverse drug reactions, Medication safety, Hospital readmission, Scoping review, General internal medicine, Risk factors, Prediction

## Abstract

**Background:**

Hospital readmissions due to medication-related problems occur frequently, burdening patients and caregivers emotionally and straining health care systems economically. In times of limited health care resources, interventions to mitigate the risk of medication-related readmissions should be prioritized to patients most likely to benefit. Focusing on general internal medicine patients, this scoping review aims to identify risk factors associated with drug-related 30-day hospital readmissions.

**Methods:**

We began by searching the Medline, Embase, and CINAHL databases from their inception dates to May 17, 2022 for studies reporting risk factors for 30-day drug-related readmissions. We included all peer-reviewed studies, while excluding literature reviews, conference abstracts, proceeding papers, editorials, and expert opinions. We also conducted backward citation searches of the included articles. Within the final sample, we analyzed the types and frequencies of risk factors mentioned.

**Results:**

After deduplication of the initial search results, 1159 titles and abstracts were screened for full-text adjudication. We read 101 full articles, of which we included 37. Thirteen more were collected via backward citation searches, resulting in a final sample of 50 articles. We identified five risk factor categories: (1) patient characteristics, (2) medication groups, (3) medication therapy problems, (4) adverse drug reactions, and (5) readmission diagnoses. The most commonly mentioned risk factors were polypharmacy, prescribing problems—especially underprescribing and suboptimal drug selection—and adherence issues. Medication groups associated with the highest risk of 30-day readmissions (mostly following adverse drug reactions) were antithrombotic agents, insulin, opioid analgesics, and diuretics. Preventable medication-related readmissions most often reflected prescribing problems and/or adherence issues.

**Conclusions:**

This study’s findings will help care teams prioritize patients for interventions to reduce medication-related hospital readmissions, which should increase patient safety. Further research is needed to analyze surrogate social parameters for the most common drug-related factors and their predictive value regarding medication-related readmissions.

**Supplementary Information:**

The online version contains supplementary material available at 10.1186/s12913-023-10028-2.

## Background

Hospital readmissions burden patients emotionally and increase healthcare systems’ economic challenges [1, 2]. One prevalent medical intervention in general internal medicine is medication therapy, with regimens often initiated or changed during a hospital stay. As a result, shortly after patients are discharged, they experience new medication-related problems (MRPs) [3–5], i.e., “event[s] or circumstance[s] involving drug therapy that actually or potentially interfere with desired health outcomes” [6].

MRPs can be either adverse drug reactions (ADRs) or medication errors (MEs). The latter encompass erroneous drug administration, incorrect dosages, unnecessary or omitted drug therapy (respectively over- or underprescribing), and non-adherence [7]. When severe, MRPs can lead to hospital readmissions. For example, too high a dose of diuretics can cause an excessive drop in blood pressure, precipitating a fall, which may require hospitalization.

Of the many thresholds used for readmission times, 30 days is the most common [8]. This is likely because the Centers for Medicare and Medicaid Services (CMS) penalize health institutions financially for higher-than-expected 30-day readmission rates [9]. Whatever the threshold, though, both to improve patient safety and reduce avoidable health care expenditures, it is vital to minimize preventable readmissions.

A substantial proportion of readmissions are for MRPs. A 2018 systematic review found that the median prevalence of medication-related readmissions (MRRs) was 21% of all readmissions [10]. Of those MRRs, 69% were deemed potentially preventable [10]. Various studies have reported MRR risk factors [11–15]. In addition to ADRs and MEs, these can include combinations of factors such as polypharmacy and increased patient age [11–15]. However, no previous study has set out to synthesize evidence on MRR risk factors by means of a literature review in a general internal medicine population as their primary objective. El Morabet et al. [10] conducted a systematic literature review to determine the prevalence and preventability of MRRs. And while they did provide a summary of reported risk factors retrospectively, they did not prospectively search for them. Another study by Linkens et al. [16] provided an overview of the literature on risk factors for medication-related admissions and MRRs. However, this study only searched one bibliographic database and did not clearly distinguish between risk factors for medication-related admissions and MRRs [16]. Additionally, both studies [10, 16] did not focus on short-term readmissions. As this knowledge gap about short-term MRRs has expensive and burdensome consequences, the purpose of the current scoping review is to provide an overview of risk factors linked to medication-related 30-day hospital readmissions in a general internal medicine department’s adult population. Identifying such risk factors is essential to helping hospitals target patients most likely to benefit from relevant interventions.

## Methods

### Study design

Combined with the Arskey and O’Malley framework [17], the Joanna Briggs Institute’s recommendations [18] for conducting scoping studies guided our literature review. For our writing, we applied the Preferred Reporting Items for Systematic reviews and Meta-Analyses extension for Scoping Reviews (PRISMA-ScR) Checklist [19]. The review protocol was not published separately, but is available upon request from the corresponding author.

### Eligibility criteria

We conducted a thorough review of the existing literature on risk factors for medication-related readmissions within 30 days of hospital discharge. The outcome of interest was 30-day readmissions, which encompassed both readmissions to the same hospital and readmissions to a different hospital, if reported accordingly. Additionally, we focused on reporting medication-related risk factors associated with these readmissions. This review included studies reporting one or more of the following factors and their association with short-term readmissions: (1) specific medications or medication groups; (2) patient characteristics or sociodemographic characteristics associated with readmissions caused by MRPs; (3) medication therapy characteristics or problems; and (4) readmission diagnoses of MRRs.

We excluded studies where no medication-related attributes are reported, where readmissions to other institutions than hospitals are analyzed, or where the time-to-readmission threshold is longer than 30 days—our timeframe of interest. Additionally, we excluded studies that analyze readmissions in patients < 18 years of age. Studies focusing on patients discharged from hospital departments other than general internal medicine, including but not limited to surgery, transplant, or oncology departments, were also excluded. Likewise, we did not consider studies that focus solely on specific patient populations (e.g., heart failure patients) or medication groups (e.g., diuretics). As we did not assess each included study’s bias risk and were interested in original research, we excluded conference abstracts, proceeding papers, editorials, expert opinions, reviews, and any papers for which full-text versions are not available. As the researchers are fluent in English, German, and French, studies in those languages were included; those published in other languages were excluded.

### Information sources, search strategy

We searched the MEDLINE (inception year: 1946), Embase (inception year: 1974), and CINAHL (inception year: 1961) databases using Ovid for the first two and EBSCO for the last. We searched the entire databases until the dates of our searches, the last of which was conducted on May 17^th^ 2022. For included studies and literature reviews with research questions similar to ours [10, 16], we also conducted manual backward citation searches.

We combined and searched subject headings (SHs) for *readmissions*, *MEs* or *ADRs*, and *risk factors* or *risk assessment*. Additionally, we searched titles and abstracts for these SHs (and synonyms), as well as *medication groups* (and synonyms) in abstracts and titles (no SHs available), combined with the index term *readmission* (and its synonyms) in abstracts and titles. All search strings for title and abstract fields were constructed to account for any alternative spellings.

The search strategy was discussed and optimized with a medical librarian. The strategies used in the individual databases are provided in Supplementary File 1. No limits were applied to the searches regarding language or date of publication. In Ovid Embase, the records were filtered to exclude conference materials. Records were deduplicated using EndNote 20 (2013, Clarivate, Philadelphia, USA) and manually—by finding and deleting duplicates—using Microsoft Excel (2016, Microsoft, Redmond, USA).

### Study selection, data extraction, and synthesis of results

Two reviewers independently screened the retrieved records’ titles and abstracts for eligibility. In a second step, potentially eligible records’ full texts were reviewed for inclusion. Disagreements at both stages were solved through personal communication.

From the included studies, one reviewer collected data, which were then verified by the second author. Relevant data items were entered to a predefined table. In a second step, this table was summarized and aggregated, where factors and certain study characteristics could be checked off, filled in with numbers or answered with dichotomous outcomes.

The first table (Supplementary File 2) presented eight data fields: publication year, country of origin, study type, study site, study population, whether readmissions' causality or preventability were analyzed, objectives, and readmission rates (if analyzed and reported). To ensure comparability across the included studies, readmission rates were calculated as percentages by dividing the absolute number of reported readmissions by the total number of studied admissions. This calculation was performed only for studies that did not already report the readmission rates as percentage values. The main part of the first table lists medication relevant factors (i.e., patient characteristics, medication group or medication therapy characteristics and problems associated with MRRs). Where medication groups or specific active substances are listed, we have added the Anatomical Therapeutic Chemical (ATC) classification system codes in square brackets. The aggregated table (Supplementary File 3) contains the following information: whether age or number of medications taken were preselected in the studied population; whether the study uses a readmission risk stratification model; whether causality and/or preventability is assessed; and follow-up time in days. Additionally, we have added a row for each factor and headed it with that factor’s name. As the studies are listed in columns, we have inserted a check-box in the corresponding cell to indicate each factor's presence. If a particular risk factor is connected with a preventable readmission, a P is included in superscript. If the study classifies ≥ 50% of readmissions including a particular risk factor as preventable, a “(P)” is added in superscript. Causality of the readmission was considered established if a validated tool like the Naranjo scale [20] or the AT-HARM 10 criteria [21] was utilized, or if physician judgment, such as through a coded ADR, was used to classify the readmission due to a medication-related problem. Conversely, no causality was assumed if the studies examined all-cause 30-day readmissions and identified, e.g., a higher prevalence of certain medication groups in the readmitted group compared to the non-readmitted group. In the all-cause readmissions, we only extracted data connected with medications (i.e., medication groups, or number of medications, being dependent on help with medications etc.). This means that in this scoping review, causality refers to whether the included studies analyzed if the identified risk factor (e.g., a certain medication) was justifiably the reason for the readmission.

## Results

**Fig. 1:**
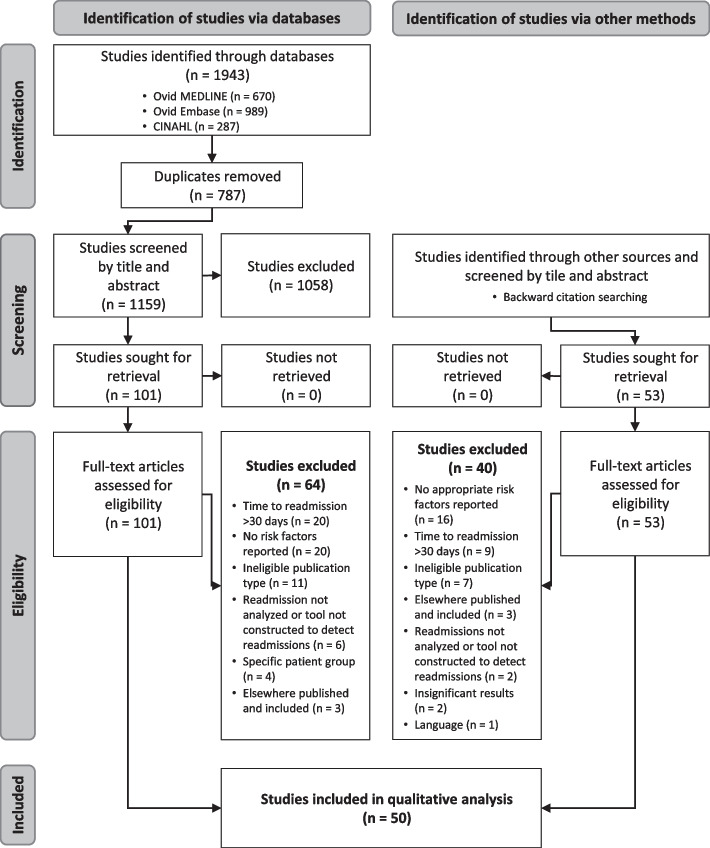
PRISMA 2020 [19] flow diagram. Abbreviations: n, number

After deduplication, we retrieved 1159 publications. We excluded 1058 studies through title and abstract screening, mostly because no risk factors for MRRs were reported or only in specific patient populations. Subsequently, we retrieved 101 full-text studies to analyze eligibility. Of these, we included 37. Through backward citation searches, we additionally retrieved 53 full texts, of which 13 proved eligible, pushing the sample size up to 50 publications. A flow diagram of the literature review process is shown in Fig. 1.

An overview of the included studies is provided in Table 1.

**Table 1:** Overview of included studies

Studies	**Uitvlugt, E. B., et al. (2021)** [14]	**Cooper, J. B., et al. (2020)** [22]	**Whitaker, A. S., et al. (2019)** [15]	**Dalleur, O., et al. (2021)** [12]	**Dreyer, R., et al. (2019)** [23]	Yam, C. H., et al. (2010) [24]	Frankl, S. E., et al. (1991) [25]	**Toh, M. R., et al. (2014) **[26]	**Willson, M. N., et al. (2014)** [27]	**Banholzer, S., et al. (2021)** [11]	**Nikolaus, T., et al. (1992)** [28]	**Ekerstad, N., et al. (2017)** [29]	**Rothwell, M., et al. (2011)** [30]
Preselection: Age	-	-	-	-	-	-	-	-	-	-	Geri	75 +	65 +
Preselection: Number of medications	-	-	-	-	-	-	-	-	-	-	-	-	3 +
Model	N	N	N	N	N	N	N	N	N	N	N	N	N
Causality (medication-related)	Y	Y	Y	Y	Y	Y	Y	Y	Y	Y	Y	Y	Y
Preventability	Y	Y	Y	Y	Y	Y	Y	N	N	N	Y	Y	Y
Follow-up time [days]	30	30	28	30	30	30	30	15	30	30	21	30	28
Country	NL	US	AU	US	ZA	CN	US	SG	US	CH	DE	SE	AU
Readmissions on total admissions [%]	-	-	-	-	10.5	16.9	12.5	-	-	-	-	24.6	-
Preventable readmissions on total admissions [%]	-	-	-	-	-	-	-	-	-	-	-	-	-
Preventable readmissions on total readmissions [%]	-	-	-	-	-	40.8	8.6	-	-	-	-	-	-
MRRs on total readmissions [%]	16	50.2	21	13.1	12.6	-	20	15.9	-	20.9	-	33.3	23
Potentially preventable MRRs on total MRRs [%]	40	57.8	59	-	11.9	-	13.8	-	-	-	-	65.6	87
Studies	**Beckner, A., et al. (2021)** [42]	Allaudeen, N., et al. (2011) [43]	Rosen, O. Z., et al. (2017) [44]	Logue, E., et al. (2016) [45]	**Lau, M. H. M., et al. (2017)** [46]	**Pavon, J. M., et al. (2014)** [47]	**Ibarra Mira, M. L., et al. (2021)** [48]	**Pereira, F., et al. (2021)** [49]	**Basnet, S., et al. (2018)** [50]	**Lohman, M. C., et al. (2017) **[51]	Schoonover, H., et al. (2014) [52]	Anderson, R. E., et al. (2016) [53]	Blanc, A. L., et al. (2019) [54]
Preselection: Age	-	-	-	-	75 +	60 +	-	65 +	65 +	65 +	50 +	65 +	-
Preselection: Number of medications	-	-	-	-	-	-	5 +	5 +	-	-	-	-	-
Model	N	N	N	N	N	N	N	N	N	N	N	N	Y
Causality (medication-related)	N	N	N	N	N	N	N	N	N	N	N	N	N
Preventability	N	N	N	N	N	N	N	N	N	N	N	N	Y
Follow-up time [days]	30	30	30	30	28	30	30	30	30	30	30	30	30
Country	US	US	US	US	CN	US	ES	CH	US	US	US	US	CH
Readmissions on total admissions [%]	-	17	14.7	14	25.5	21	25.6	7.8	16	14.6	-	21.8	-
Preventable readmissions on total admissions [%]	-	-	-	-	-	-	-	-	-	-	-	-	7.5
Preventable readmissions on total readmissions [%]	-	-	-	-	-	-	-	-	-	-	-	-	-
MRRs on total readmissions [%]	-	-	-	-	-	-	-	-	-	-	-	-	-
Potentially preventable MRRs on total MRRs [%]	-	-	-	-	-	-	-	-	-	-	-	-	-
Studies	Witherington, E. M., et al. (2008) [31]	**Weir, D. L., et al. (2020)** [32]	**Glans, M., et al. (2021)** [13]	**Schwab, C., et al. (2018)** [33]	**Barnett, N. L., et al. (2017)** [34]	**Koekkoek, D., et al. (2011)** [35]	**Balla, U., et al. (2008)** [36]	Feigenbaum, P., et al. (2012) [37]	**Classen, D. C., et al. (2010)** [38]	**Porter, M., et al. (2019)** [39]	**Wetherell, M., et al. (2017)** [40]	**Aljishi, M., et al. (2014)** [41]	
Preselection: Age	75 +	65 +	65 +	75 +	-	-	-	-	-	-	-	-	
Preselection: Number of medications	-	1 +	-	-	-	-	-	-	-	-	-	-	
Model	N	N	N	N	Y	N	N	N	N	N	N	N	
Causality (medication-related)	Y	Y	Y	Y	Y	N	N	N	N	N	N	N	
Preventability	Y	N	N	N	Y	Y	Y	Y	N	N	N	N	
Follow-up time [days]	28	30	30	30	30	21	30	30	30	30	30	30	
Country	GB	CA	SE	FR	GB	US	IL	CA	US	US	US	NZ	
Readmissions on total admissions [%]	-	35.4	-	6.4	16	-	14.1	-	-	46.2	10.7	4.97	
Preventable readmissions on total admissions [%]	-	-	-	-	-	-	-	-	-	-	-	-	
Preventable readmissions on total readmissions [%]	-	-	-	-	-	-	-	47	-	-	-	-	
MRRs on total readmissions [%]	38	-	40	11.4	-	-	-	-	-	-	-	-	
Potentially preventable MRRs on total MRRs [%]	61	-	-	-	1.7	-	-	-	-	-	-	-	
Studies	McAuliffe, L. H., et al. (2018) [55]	**Sorensen, A., et al. (2021)** [56]	**Fung, L., et al. (2020)** [57]	**Trautwein, M., et al. (2020)** [58]	**Criddle, D. T., et al. (2021)** [59]	**Picker, D., et al. (2015)** [60]	**Nguyen, H. L., et al. (2022)** [61]	Dorajoo, S. R., et al. (2017) [62]	**SanFilippo, S., et al. (2021)** [63]	**Leffler, M. E., et al. (2019)** [64]	**Sieck, C., et al. (2019)** [65]	Meldon, S. W., et al. (2003) [66]	
Preselection: Age	-	-	-	-	-	-	-	-	65 +	65 +	65 +	65 +	
Preselection: Number of medications	-	-	-	-	-	-	-	-	5 +	-	-	-	
Model	Y	Y	Y	Y	Y	Y	Y	Y	Y	Y	Y	Y	
Causality (medication-related)	N	N	N	N	N	N	N	N	N	N	N	N	
Preventability	Y	N	N	N	N	N	N	N	N	N	N	N	
Follow-up time [days]	30	30	30	30	30	30	30	15	30	30	30	30	
Country	US	US	US	US	AU	US	US	SG	US	US	US	US	
Readmissions on total admissions [%]	-	21.4	13	12.6	25.9	20.8	15	-	15	-	-	31.9	
Preventable readmissions on total admissions [%]	14.8	-	-	-	-	-	-	-	-	-	-	-	
Preventable readmissions on total readmissions [%]	-	-	-	-	-	-	-	-	-	-	-	-	
MRRs on total readmissions [%]	-	-	-	-	-	-	-	-	-	-	-	-	
Potentially preventable MRRs on total MRRs [%]	-	-	-	-	-	-	-	-	-	-	-	-	

Studies identified through databases are depicted in bold, those identified through citation searches in regular type

*Abbreviations*: *N* No, *Y* Yes, *Geri* Geriatric population, *MRR* Medication-related readmissions, *NL* Netherlands, *US* United States, *AU* Australia, *ZA* South Africa, *CN* China, *SG* Singapore, *CH* Switzerland, *DE* Germany, *SE* Sweden, *GB* United Kingdom, *IL* Israel, *CA* Canada, NZ New Zealand, *ES* Spain

Studies identified through database searches are shown in bold; those identified through citation searches are in regular text. Of the included studies, 18 assess causality [11–15, 22–34], particularly specifying whether the readmissions were caused by MRPs. The other 32 [35–66] examine medication-associated factors (e.g., whether readmitted patients were more likely to have had polypharmacy), but do not establish whether the readmissions were caused by MRPs. Fourteen studies describe risk prediction models [34, 54–66]. In 19, the study population was preselected based on advanced age (most often ≥ 65 years (*n* = 11)) and/or number of medications taken [13, 28–33, 46–53, 63–66]. Specifically, 14 preselected their participants based on age [13, 28, 29, 31, 33, 46, 47, 50–53, 64–66], one based on number of medications [48], and four based on a combination of age and number of medications [30, 32, 49, 63]. The readmissions’ preventability was analyzed in 17 publications [12, 14, 15, 22–25, 28–31, 34–37, 54, 55]. The most common readmission time threshold (*n* = 42, 84%) is 30 days [11–14, 22–25, 27, 29, 32–34, 36–45, 47–61, 63–66], followed by 28 days (*n* = 4, 8%) [15, 30, 31, 46]. Two studies each examine 21-day [28, 35] and 15-day readmissions (4% each) [26, 62]. Most studies (*n* = 27) were conducted in the US [12, 22, 25, 27, 35, 38–40, 42–45, 47, 50–53, 55–58, 60, 61, 63–66]. The comprehensive table with all extracted characteristics can be found in Supplementary File 2.

### Primary outcomes

For the sake of clarity, only MRR risk factors mentioned by at least three included studies are presented in this manuscript. A table of all factors, indicating which studies reported them, can be found in Supplementary File 3. The results’ section is divided into three parts: in the first and main part, we include the findings of studies assessing the causality of the readmissions, in the second part, we highlight differences, if the findings are combined with non-causality studies; and in the third and last part, we provide an insight into medication-related risk factors for preventable readmissions.

### Patient characteristics – studies establishing causality

Twelve of the included 18 studies, that assessed causality, mentioned medication-relevant patient characteristics [13–15, 26–34]. Regarding studies that preselected their study populations based on older age or higher numbers of medications, we included these criteria as risk factors. Advanced age (most often ≥ 65 years) is the patient characteristic that most commonly accompanies MRRs (mentioned in eight studies) [13, 15, 28–33]. Seven mention polypharmacy and/or specific number of drugs as risk factors [13, 15, 26, 27, 29, 33, 34]. Three causality studies define the number of medications considered polypharmacy, the most common threshold is ≥ 10 (*n* = 2) [13, 29]. Three studies highlight the number of changes in patients’ medication regimens during their initial hospital stay as factors associated with increased MRR rates [13, 14, 34].

### Medication groups – studies establishing causality

Ten studies mention specific medication groups that caused readmissions [11, 12, 14, 22, 23, 25, 28–30, 34]. Antithrombotics are mentioned most frequently (*n* = 9) [11, 12, 14, 22, 23, 25, 29, 30, 34], especially anticoagulants (*n* = 7) [11, 22, 23, 25, 29, 30, 34]. These are followed by antidiabetics (*n* = 8) [11, 12, 14, 22, 25, 28, 30, 34], particularly insulin [11, 12, 14, 22, 25, 28, 30, 34]. Diuretics were also mentioned eight times [11, 12, 14, 22, 25, 29, 30, 34]. Antibacterials (*n* = 7) [11, 12, 14, 22, 25, 29, 30] and opioid analgesics (*n* = 6) [11, 12, 14, 25, 29, 34] were also frequently noted. An exhaustive list of the medication groups mentioned by at least three of the included studies is shown in Table 2. In Table 2, ten studies [11, 12, 14, 22, 23, 25, 28–30, 34] assessed causality, whereas the remaining 11 studies [35, 38, 43, 47, 49, 54, 57, 59, 61, 64, 65] investigated all-cause readmissions and reported that particular medication groups were associated with a higher readmission rate.

**Table 2:** Medication groups associated with readmissions

**Studies**	**Uitvlugt, E. B., et al. (2021)** [14]	**Cooper, J. B., et al. (2020)** [22]	**Dalleur, O., et al. (2021)** [12]	**Dreyer, R., et al. (2019)** [23]	**Frankl, S. E., et al. (1991)** [25]	**Banholzer, S., et al. (2021)** [11]	**Nikolaus, T., et al. (1992)** [28]	**Ekerstad, N., et al. (2017)** [29]	**Rothwell, M., et al. (2011)** [30]	**Barnett, N. L., et al. (2017)** [34]	**Koekkoek, D., et al. (2011)** [35]
Laxatives [A06A]	X		X								
Antidiabetics [A10]	X	X	X		X	X	X		X	X	
Insulin [A10A]	X	X	X		X	X			X	X	
Oral antidiabetics [A10B]	X					X	X			X	
Mineral supplements [A12]	X				X	X					
Antithrombotic agents [B01]	X	X	X	X	X	X		X	X	X	X
Anticoagulants [B01AA, B01AB, B01AF, B01AX]		X		X	X	X		X	X	X	X
Vitamin K antagonists [B01AA]		X		X	X	X		X	X		
Warfarin [B01AA03]		X		X	X			X	X		
Heparin group [B01AB]						X			X		
Antiplatelets excl. heparins [B01AC]		X			X	X		X		X	
Acetyl salicylic acid [B01AC06]		X			X			X			
Cardiac glycosides [C01AA]	X				X			X			
Digoxin [C01AA05]					X			X			
Antiarrhythmic agents [C01B]			X		X						
Vasodilator [C01D, C04]	X		X						X		
Antihypertensives [C02, C03, C07, C08, C09]	X	X	X		X	X		X	X	X	
Diuretics [C03]	X	X	X		X	X		X	X	X	
Saluretics [C03A, C03B, C03C, C03D, C03E]						X		X			
Sympatholytics [C02A, C02B, C02C, C07]	X		X			X		X	X	X	
Beta-blocking agents [C07A]	X		X			X		X	X	X	
Calcium channel blocker [C08]	X				X	X		X	X		
Agents acting on renin-angiotensin system [C09]	X	X	X			X		X	X		
ACE inhibitors [C09AA]		X				X			X		
Corticosteroids [H02]	X		X		X	X				X	
Antibacterial for systemic use [J01]	X	X	X		X	X		X	X		
Antineoplastic agents [L01]			X		X	X					
Immunosuppressants [L04]	X		X		X	X			X	X	
Analgesics [N02]	X		X		X	X		X	X	X	
Opioids and drugs used against addiction [N02A]	X		X		X	X		X		X	
Antiepileptics [N03A]	X					X		X			
Hydantoin derivatives [N03AB]						X					
Carboxamide derivatives [N03AF]						X		X			
Psycholeptics [N05]					X	X			X	X	
Hypnotics and sedatives [N05C]					X	X			X		
Anxiolytics [N05B]										X	
Benzodiazepine based tranquillizers [N05BA/N05CD/N05CF]						X			X	X	
Benzodiazepine derivatives [N05BA]						X					
Psychoanaleptics [N06]		X				X		X	X		
Antidepressants [N06A]		X				X		X	X		
NSAIDs [M01A]						X				X	
Medications for obstructive airway diseases (asthma/COPD) [R03]	X	X			X	X					

**Studies**	**Classen, D. C., et al. (2010)** [38]	**Allaudeen, N., et al. (2011)** [43]	**Pavon, J. M., et al. (2014)** [47]	**Pereira, F., et al. (2021)** [49]	**Blanc, A. L., et al. (2019)** [54]	**Fung, L., et al. (2020)** [57]	**Criddle, D. T., et al. (2021)** [59]	**Nguyen, H. L., et al. (2022)** [61]	**Leffler, M. E., et al. (2019)** [64]	**Sieck, C., et al. (2019)** [65]	**Quantity mentioned**
Laxatives [A06A]				X							**3**
Antidiabetics [A10]				X		X	X	X	X	X	**14**
Insulin [A10A]							X	X	X	X	**11**
Oral antidiabetics [A10B]										X	**5**
Mineral supplements [A12]											**3**
Antithrombotic agents [B01]	X					X	X	X		X	**15**
Anticoagulants [B01AA, B01AB, B01AF, B01AX]	X					X	X	X			**12**
Vitamin K antagonists [B01AA]								X			**7**
Warfarin [B01AA03]								X			**6**
Heparin group [B01AB]	X					X					**4**
Antiplatelets excl. heparins [B01AC]						X				X	**7**
Acetyl salicylic acid [B01AC06]										X	**4**
Cardiac glycosides [C01AA]							X			X	**5**
Digoxin [C01AA05]							X			X	**4**
Antiarrhythmic agents [C01B]							X				**3**
Vasodilator [C01D, C04]											**3**
Antihypertensives [C02, C03, C07, C08, C09]				X			X	X			**11**
Diuretics [C03]				X			X	X			**11**
Saluretics [C03A, C03B, C03C, C03D, C03E]								X			**3**
Sympatholytics [C02A, C02B, C02C, C07]				X							**7**
Beta-blocking agents [C07A]				X							**7**
Calcium channel blocker [C08]							X				**6**
Agents acting on renin-angiotensin system [C09]											**6**
ACE inhibitors [C09AA]											**3**
Corticosteroids [H02]		X	X						X		**8**
Antibacterial for systemic use [J01]											**7**
Antineoplastic agents [L01]							X				**4**
Immunosuppressants [L04]						X	X				**8**
Analgesics [N02]		X	X		X	X	X	X	X	X	**15**
Opioids and drugs used against addiction [N02A]		X	X		X	X	X	X	X	X	**14**
Antiepileptics [N03A]			X			X	X	X	X		**8**
Hydantoin derivatives [N03AB]							X	X			**3**
Carboxamide derivatives [N03AF]							X				**3**
Psycholeptics [N05]			X	X			X	X			**8**
Hypnotics and sedatives [N05C]											**3**
Anxiolytics [N05B]			X				X				**3**
Benzodiazepine based tranquillizers [N05BA/N05CD/N05CF]			X				X				**5**
Benzodiazepine derivatives [N05BA]			X				X				**3**
Psychoanaleptics [N06]			X								**5**
Antidepressants [N06A]			X								**5**
NSAIDs [M01A]							X				**3**
Medications for obstructive airway diseases (asthma/COPD) [R03]							X				**5**

ATC code(s) of the medications are in square brackets

*Abbreviations*: *ACE* Angiotensin converting enzyme, *COPD* Chronic obstructive pulmonary disease, *NSAIDs* non-steroidal anti-inflammatory drugs

### Medication groups associated with adverse drug reactions leading to readmissions – studies establishing causality

Ten studies describe ADRs leading to rehospitalizations [11, 13, 15, 22–25, 28, 29, 31]. Results indicate that ADRs involving antithrombotic agents, especially vitamin K antagonists, which are anticoagulants, were most often associated with readmissions due to ADRs [11, 23, 25, 29]. Adverse reactions to antibacterial agents are also noted in three of the included studies [11, 25, 29].

### Medication therapy problems excluding adverse drug reactions – studies establishing causality

Fifteen records study medication therapy problems other than ADRs as reasons for readmissions [12–15, 22–26, 28–31, 33, 34]. MRR-associated medication therapy problems are most commonly adherence issues (*n* = 13) [12–15, 22–26, 28, 30, 31, 34] and prescribing problems (*n* = 12) [12–15, 22, 24–26, 29–31, 33]. If a reason is given for suboptimal medication adherence, it is usually either erratic use [13, 15] or difficulty using a specific dosage form [13, 15, 22]. The most relevant prescribing problem is misprescribing (*n* = 10) [12–15, 22, 24–26, 30, 33], most commonly either underdose (*n* = 6) [12, 13, 15, 22, 26, 30] or overdose (*n* = 6) [12, 15, 22, 25, 26, 33], drug-drug interactions (*n* = 4) [12, 13, 24, 25], or suboptimal medication selection [15, 22, 30]. Another misprescribing problem mentioned in three studies was the prescription of a contraindicated medication [13, 22, 25]. Misprescribing problems are followed by underprescribing (*n* = 9) [12–15, 22, 24, 29–31]. Overprescribing is a risk factor mentioned by five studies [12, 14, 15, 22, 31]; however, fewer MRRs are attributed to it compared to underprescribing. Insufficient ambulatory monitoring of medication therapy was mentioned six times [12–14, 22, 30, 31], whereas transition of care errors were mentioned five times [14, 25, 28, 30, 31].

### Readmission diagnoses – studies establishing causality

Seven publications report MRR diagnoses [12, 25, 26, 28–30, 33]. Hypotension is the readmission diagnosis encountered most often (*n* = 5) [12, 26, 29, 30, 33]. This is followed by myeolsuppression (including diagnoses of anemia, neutropenia, and/or thrombocytopenia) [12, 25, 26, 30], constipation [12, 26, 29, 30], hypoglycemia [12, 26, 28, 30], and bleeding [12, 25, 29, 30], each mentioned by four studies. Arrhythmia (including atrial fibrillation) or QT prolongation was encountered three times [12, 29, 30]. In four, suboptimal patient monitoring or education about antithrombotic agents led to bleeding [12, 25, 29, 30] and overprescribing or overdose of antihypertensives to hypotension [12, 29, 30, 33]. Allergy or rash [12, 25, 26], infection [12, 29, 30], falls [29, 30, 33], or heart failure [12, 29, 30] were each named in three studies as reasons behind MRRs.

### Differences in medication-related risk factors between studies establishing causality and studies that do not

With regard to patient characteristics, the results did not differ significantly when adding studies not establishing causality to the analysis. Twenty-four studies, not establishing causality, reported on medication-related patient characteristics that were linked with a higher rate of 30-day all-cause readmissions [35, 36, 39–41, 45, 46, 48–53, 55–60, 62–66]. Of these, 19 studies showed a higher readmission rate in patients with polypharmacy [36, 39–41, 45, 48–50, 55–60, 62–66]. Five studies highlight the prescription of potentially inappropriate medications (PIMs) [32, 33, 46, 51, 60] and an additional one the number of medication changes [35] as factors associated with increased readmission rates. Finally, three studies correlate increased readmission rates with high medication regimen complexity index (MRCI) scores [34, 52, 60].

In line with patient characteristics, when considering the 11 non-causality studies that reported on medication groups associated with readmissions [35, 38, 43, 47, 49, 54, 57, 59, 61, 64, 65], the results did not change much: most frequently mentioned medication groups were still very prominent. Only antibacterials were not mentioned in non-causality studies. The detailed results for all the medication groups are shown in Table 2.

Four non-causality studies were included that reported on medication therapy problems excluding ADRs [36, 37, 42, 44]. ADR-specific analyses were not included in non-causality studies. Readmission diagnoses in connection with medications associated with readmission were cited in one non-causality study [61]. Therefore, the results described above only change slightly, and we refer to the Supplementary File 3 for details.

### Readmission rates and preventability

All-cause readmission rates are reported by 29 of the included studies (see Table 1), with a mean rate of 18.34 ± 8.78%. When three publications assessed what proportion of these readmissions were potentially preventable, they arrived at a cross-study mean of 32.13 ± 16.83%. Two also analyzed the rate of potentially preventable readmissions on total admissions (including readmissions), finding a mean of 11.15 ± 3.65%. The percentage of total readmissions classed as MRRs was analyzed by 13 studies, with an overall mean of 24.26 ± 11.76%; and across nine studies, the mean percentage of all MRRs that were potentially preventable is 44.2 ± 27.42%.

Readmission preventability is investigated directly in 14 studies [12, 14, 15, 22–25, 28–31, 35–37]. Three others describe risk prediction models targeted at preventable readmissions [34, 54, 55]. Regarding our listing of the various studies’ factors (Table 3), as one of those predictive models—by Barnett et al. [34]—does not specify which factors are predictive of preventable MRRs (i.e., no validation), we did not classify any of their listed factors as preventable. Similarly, as Dreyer et al. [23] could not identify medication-related risk factors associated with readmission preventability in ≥50% of cases, we also omitted these data. Respective drug groups, MRPs, and readmission diagnoses associated with preventable readmissions are tabulated in Table 3. Ten of the included studies assessed causality [12, 14, 15, 22, 24, 25, 28–31] of the readmissions, whereas four of them did not [35–37, 54].

**Table 3:** Factors associated with preventable readmissions

**Studies**	**Uitvlugt, E. B., et al. (2021) ** [14]	**Cooper, J. B., et al. (2020) ** [22]	**Whitaker, A. S., et al. (2019) ** [15]	**Dalleur, O., et al. (2021) ** [12]	**Yam, C. H., et al. (2010) ** [24]	**Frankl, S. E., et al. (1991) ** [25]	**Nikolaus, T., et al. (1992) ** [28]	**Ekerstad, N., et al. (2017) ** [29]	**Rothwell, M., et al. (2011) ** [30]	**Witherington, E. M., et al. (2008) ** [31]	**Koekkoek, D., et al. (2011) ** [35]	**Balla, U., et al. (2008) ** [36]	**Feigenbaum, P., et al. (2012) ** [37]	**Blanc, A. L., et al. (2019) ** [54]	**Quantity mentioned**
**Drug groups**															
Antidiabetics [A10]	X^P^			X^P^					X^P^						**3**
Insulin [A10A]	X^P^			X^P^					X^P^						**3**
Antithrombotic agents [B01]	X^P^			X^P^		X^P^			X^P^		X^P^				**5**
Anticoagulants [B01AA, B01AB, B01AF, B01AX]						X^P^			X^P^		X^P^				**3**
Antiplatelets excl. heparins [B01AC]	X^P^	X^(P)^				X^P^									**3**
Acetyl salicylic acid [B01AC06]	X^P^	X^(P)^				X^P^									**3**
Cardiac glycosides [C01AA]	X^P^					X^P^		X^P^							**3**
Vasodilator [C01D, C04]	X^P^			X^P^					X^P^						**3**
Antihypertensives [C02, C03, C07, C08, C09]	X^P^	X^(P)^		X^P^					X^P^						**4**
Diuretics [C03]	X^P^	X^(P)^		X^P^					X^P^						**4**
Sympatholytics [C02A, C02B, C02C, C07]	X^P^			X^P^					X^P^						**3**
Beta-blocking agents [C07A]	X^P^			X^P^					X^P^						**3**
Agents acting on renin-angiotension system [C09]	X^P^	X^P^		X^P^					X^P^						**4**
Antibacterial for systemic use [J01]	X^P^	X^(P)^		X^P^					X^P^						**4**
Analgesics [N02]	X^P^			X^P^					X^P^					X^P^	**4**
Opioids and drugs used against addiction [N02A]	X^P^			X^P^										X^P^	**3**
**Medication-related problems except adverse drug reactions**															
Prescribing problems	X^P^	X^(P)^	X^(P)^	X^P^	X^P^	X^P^		X^P^	X^P^	X^P^		X^P^			**10**
Overprescribing ("no indication")	X^P^		X^P^	X^P^						X^P^					**4**
Underprescribing ("indication but no drug")	X^P^	X^(P)^	X^(P)^	X^P^	X^P^			X^P^	X^P^	X^P^		X^P^			**9**
Misprescribing ("indication but suboptimal drug prescribing)	X^P^	X^(P)^	X^P^	X^P^	X^P^	X^P^			X^P^			X^P^			**8**
Wrong dose	X^P^	X^(P)^	X^P^	X^P^					X^P^						**5**
Overdose		X^(P)^	X^P^	X^P^											**3**
Underdose		X^(P)^	X^P^	X^P^					X^P^						**4**
Suboptimal therapy/drug selection			X^P^						X^P^			X^P^			**3**
Adherence issues	X^P^	X^P^	X^(P)^	X^P^	X^P^		X^(P)^		X^P^	X^P^					**8**
Insufficient ambulatory monitoring	X^P^	X^(P)^		X^P^					X^P^	X^P^					**5**
Transition errors	X^P^					X^P^			X^P^	X^P^			X^P^		**5**
Incomplete medication discharge information									X^P^	X^P^			X^P^		**3**
**Adverse drug reactions**		X^P^	X^(P)^							X^P^					**3**
**Readmission Diagnosis**															
Arrhythmia (including atrial fibrilation) or QT prolongation				X^P^				X^P^	X^P^						**3**
Heart failure				X^P^				X^P^	X^P^						**3**
Infection				X^P^				X^P^	X^P^						**3**

ATC code(s) of the medications in square brackets

X^(P)^ is used if preventable and non-preventable readmissions were analyzed together. If at least 50% of the readmissions were deemed preventable, a X^(P)^ is charted for the respective risk factor

In summary, the most prevalent factors associated with preventable MRRs are *prescribing problems*, (most often *underprescribing*), *adherence issues*, *insufficient ambulatory monitoring*, and *transfer of care-errors*, e.g., incomplete medication discharge information. The drug group most often associated with preventable MRRs is *antithrombotic agents*.

## Discussion

To our knowledge, this is the first review to assess 30-day MRRs in adult patients in general internal medicine. We grouped our findings into five categories: *patient characteristics*, *medication groups*, *MRPs*, *ADRs*, and *readmission diagnoses*. These categories reflect the heterogeneity of the included studies. Several articles report MRPs, including ADRs, suggesting that the researchers were specifically checking whether the associated readmissions were caused by MRPs. Others describe or validate readmission risk prediction models. Where these models identify risk factors associated with medications, we have included those factors in our analysis. Much of a risk prediction model’s development is based on the idea that predictive factors—e.g., *number of medications* or the prescription of certain *medication groups*—must be entered in electronic medical records in a standardized way [67]. Furthermore, our analysis entails studies that examined all-cause readmissions. In those cases, we also extracted medication-related factors. Additionally, the diversity of the categories reflects readmissions’ tendency to be multifactorial and complex.

Patient characteristics contributing to MRRs were *older age* (most often ≥ 65 years), *polypharmacy*, *number of medication changes at initial hospital stay*, *being prescribed PIMs*, having *a higher MRCI*, and *being help-dependent in the home environment*. The medication groups most often associated with MRRs were *antithrombotics*, *opioid analgesics*, *insulin*, and *diuretics*. The most common diagnoses for MRRs were *bleeding*, *constipation*, *hypoglycemia*, and *hypotension*. These can be connected to the identified medication groups. As an example, as antithrombotic agents reduce the formation of thrombi, they can also lead to bleeding. And as opioids commonly induce constipation, laxatives should be prescribed along with them [68, 69]. In the case of insulin, an injection at too high a dose, e.g., due to non-adherence with mealtimes or prescription errors, this may result in severe hypoglycemia, often requiring hospitalization. Lastly, especially in older adults with restricted sodium intake, diuretics can lead to volume depletion, leading to hypotension.

Although ADRs were often mentioned, MEs were also found. These include adherence problems and prescribing problems, with underprescribing found to be the most prevalent regarding MRRs. Misprescribing and overprescribing also caused MRRs in the analyzed studies. Another risk factor was suboptimal transition from an acute care setting to an ambulatory one. Compared to MEs, ADR rates tend to be higher in the literature. However, measurement bias may amplify ADRs’ prevalence, i.e., because ADRs are easier to detect and are recorded in a more standardized manner, they are more likely both to be detected and to be recorded recognizably. In many countries, including the USA, where most of the included studies originate, diagnoses are recorded using the International Classification of Diseases (ICD) codes. As ADRs can be reported by ICD codes, they are relatively readily extracted from electronic health records for retrospective studies [70–74]. MEs are not captured in this standardized manner, and their endpoints often lead medical professionals to mistake them for ADRs. For example, a prescription error that leads to an opioid analgesic overdose also becomes an ADR. Therefore, when taking measures to reduce MRRs, the promotion of medication adherence, education and monitoring, and the improvement of transition-of-care processes are all important. The identified risk factors can be used by clinicians to prioritize patients for measures to reduce MRRs. These could include a combination of medication reviews, medication reconciliation, education, and interprofessional transition-of-care programs, as a meta-analysis has proven them effective at reducing readmissions [75].

Additionally, we summarized risk factors for preventable MRRs. Considering the preventability definitions' and study designs' high levels of heterogeneity, these results should be interpreted with caution. As expected, the results indicate that, when MRPs such as prescribing problems, adherence issues, and transfer-of-care errors were assessed, they were almost always deemed preventable. Some studies also found ADRs preventable, e.g., an allergic drug reaction in a patient known to be allergic to that drug—which could also qualify as a preventable ME (misprescribing). The drug groups most often associated with preventable MRRs were *antithrombotics*, *antihypertensives*, *analgesics*, especially *opioids*, and *antibacterial agents*. This is partly reflected in the readmission diagnoses, of which *arrhythmia* (often an indication for antithrombotics in case of atrial fibrillation), *heart failure* (indication for antihypertensives, other cardiovascular drugs), and *infection* (indication for antibacterial agents) were most often associated with preventable MRRs. Therefore, patients receiving the respective drug groups or diagnoses could be prioritized for interventions to avoid readmissions.

Our findings align with those of two reviews that dealt with MRRs, but with slightly different primary objectives [10, 16]. Both reviews mentioned the Charlson Comorbidity Index [10, 16]. A higher comorbidity index is associated with polypharmacy and higher MRCIs [76, 77]—both of which are identified in our results as risk factors. Both studies noted links between specific drug groups with MRRs [10, 16], all of which are included in our results. Linkens et al. [16] also correlated adherence problems, transition of care issues, falls, and weight loss with MRRs. Our findings are similar but more detailed, mainly because our primary objectives focused specifically on risk factors for MRRs.

While our study sample provides reasonably detailed findings on physical contributors to rehospitalizations, social determinants of health (SDH) are, in our opinion, underrepresented not only here but throughout the literature. These include nutritional status [78], living arrangements [79–81], and discharge against medical advice [81], all of which correlate well with all-cause readmissions. Living in a nursing home [79, 81] or a poor neighborhood [81], or depending entirely on a state pension [81] are also associated with a higher readmission risk. Another cause for concern is a lower level of social support: unmarried, widowed, or homeless patients are more likely to be readmitted [80, 81].

The LACE index [82] and the HOSPITAL score [83] are prediction models for all-cause readmissions. The LACE index looks at length of stay (L), acuity of admission (A), comorbidity (C), and emergency department use in the previous 6 months (E); and the HOSPITAL score focuses on low hemoglobin level at discharge (H), discharge following oncological treatment (O), low sodium level (S), procedures received during hospital stay (any ICD-9 coded procedures) (P), index admission data: urgent/emergency (non-elective) (IT), number of admissions over the previous year (A), and length of stay ≥ 5 days (L) [82, 83].

Adding SDHs to the LACE index improved its predictivity [84]. However, adding them to the HOSPITAL score yielded conflicting results: in one study, the HOSPITAL c-statistic improved [85]; in another it did not change significantly [86]. This could be because the HOSPITAL score's contributing risk factors already reflect the effects of SDHs [86]. A subgroup analysis showed improved predictive power in older patients [84, 85].

Ultimately, as few of our included studies report on SDHs, their roles, if any, remain unclear. However, we hypothesize that they contribute importantly to MRRs.

For example, the clear link between SDHs and medication adherence [87–90], a critical risk factor for MRRs, is congruent with our hypothesis. We strongly suspect that SDHs are underrepresented in our findings, not because they are less important than other demographic risk factors, but because data relating to them are not readily available in electronic health records. Therefore, we suggest more detailed research to investigate SDHs’ influence on MRRs.

This scoping review has several limitations: Most notably, as we did not assess the risk of bias in the included studies, we may have included substandard research. We tried to minimize this limitation by excluding articles published without peer-review. Conversely, it is possible that excluding “grey” literature—a conscious publication bias—also limited the quality of our results. To mitigate the risk of omissions, we performed backward citation searches. And to optimize database record queries’ specificity, we added terms to our search strings representing risk factors or medication groups. One conceivable result would be that studies only mentioning very specific associations, e.g., between *polypharmacy* and *readmission,* would have been left out. Still, considering that the studies included through citation searches had no significant effects on the results, we are confident that, for the purposes of this scoping review, we identified the most pertinent risk factors. In other words, our findings regarding risk factors provide a useful starting point to prioritize patients for MRR reduction measures.

## Conclusions

This scoping review summarizes potentially MRR–indicative data identified in 50 included studies. These can be categorized as *patient characteristics*, *medication groups*, *MRPs and ADRs*, and *readmission diagnoses*. Our analyses indicate that patients who have polypharmacy and are help-dependent, prescribed PIMs, older (most often mentioned threshold ≥ 65 years), and/or received medication changes during their initial hospitalization are at higher risk of experiencing MRRs. Given the prevalence of these risk factors among frail older adults, our findings highlight the importance of targeted interventions, such as medication reviews and deprescribing unnecessary medications, for this vulnerable population. Numerous medication groups appear to be associated with MRRs, most commonly *antithrombotic agents*, *opioid analgesics*, *insulin*, and *diuretics*. The leading MRPs resulting in MRRs are *non-adherence*, *prescribing problems*, and *suboptimal transition of care*. As all of these are MEs, all are also potentially preventable. In health care settings with limited resources, these risk factors can serve as risk indicators, allowing care teams to prioritize particularly vulnerable patients for interventions aimed at reducing preventable MRRs. And finally, one question that warrants consideration is that of whether certain social factors such living situations are predictive of some types of MRPs.

### Supplementary Information

**Additional file 1.** 

**Additional file 2.**

**Additional file 3.**

## Data Availability

All data generated for or analyzed in this study is published in this article and its supplementary information.

## Abbreviations

ADR: Adverse drug reaction
ATC: Anatomical Therapeutic Chemical
ICD: International Classification of Diseases
MEs: Medication errors
MRCI: Medication regimen complexity index
MRP: Medication-related problem
MRR: Medication-related readmission
PIM: Potentially inappropriate medication
SDH: Social determinants of health
SHs: Subject headings
