# Supplementary material for: Risk factors for medication-related short-term readmissions in adults – a scoping review
Source: BMC Health Serv Res. 2023 Sep 28;23:1037. doi: 10.1186/s12913-023-10028-2 (PMC10536731; doi:10.1186/s12913-023-10028-2)
Supplement: Supplementary file 1 — Additional file 1. [file 12913_2023_10028_MOESM1_ESM.pdf]

Ovid Medline

#	Search string	Results (17th May 2022)
1	Patient Readmission/	21,526
2	(Readmission? or Re-admission? or Readmit$ or Re-admit$ or Rehospitali$ or Re-hospitali$).ti,ab.	49,720
3	1 or 2	54,303
4	"Drug-Related Side Effects and Adverse Reactions"/	35,851
5	Medication Errors/	14,058
6	((Drug? or medication? or medicine?) adj3 (related or associated)).ti,ab.	75,925
7	(Adverse adj1 (drug or medication or medicine) adj1 (reaction? or event? or effect? or outcome?)).ti,ab.	23,609
8	(side effect? or medication error? or ADR? or ADE?).ti,ab.	303,472
9	((problem? or issue?) adj3 (drug? or medication?)).ti,ab.	16,834
10	4 or 5 or 6 or 7 or 8 or 9	428,907
11	Risk Factors/	923,134
12	Risk Assessment/	299,472
13	(factor? or characteri$ or reason? or pattern? or predictor? or attribute? or incident?).ti,ab.	8,104,729
14	((predict$ or screening or risk) adj2 (model? or tool? or score? or assessment?)).ti,ab.	311,184
15	11 or 12 or 13 or 14	8,739,368
16	(((drug or medication or medicine) adj (group? or class or classes)) or ((causative or causing) adj (drug? or medication? or medicine?))).ti,ab.	16,013
17	Patient Readmission/	21,526
18	(Readmission? or Re-admission? or Readmit$ or Re-admit$ or Rehospitali$ or Re-hospitali$).ti,ab.	49,720
19	17 or 18	54,303
20	3 and 10 and 15	596
21	16 and 19	81
22	20 or 21	**670**

Execute search

Ovid Embase

#	Search String	Results (17th May 2022)
1	Hospital Readmission/	83,277
2	(Readmission? or Re-admission? or Readmit$ or Re-admit$ or Rehospitali$ or Re-hospitali$).ti,ab.	92,114
3	1 or 2	110,868
4	Adverse Drug Reaction/	264,913
5	exp Medication Error/	21,557
6	Side Effect/	338,433
7	((Drug? or medication? or medicine?) adj3 (related or associated)).ti,ab.	114,992
8	(Adverse adj1 (drug or medication or medicine) adj1 (reaction? or event? or effect? or outcome?)).ti,ab.	38,757
9	(side effect? or medication error? or ADR? or ADE?).ti,ab.	450,294
10	((problem? or issue?) adj3 (drug? or medication?)).ti,ab.	25,443
11	4 or 5 or 6 or 7 or 8 or 9 or 10	963,697
12	Risk Factor/	1,208,356
13	Risk Assessment/	664,209
14	(factor? or characteri$ or reason? or pattern? or predictor? or attribute? Or incident?).ti,ab.	10,292,930
15	((predict$ or screening or risk) adj2 (model? or tool? or score? or assessment?)).ti,ab.	435,910
16	12 or 13 or 14 or 15	11,142,388
17	(((drug or medication or medicine) adj (group? or class or classes)) or ((causative or causing) adj (drug? or medication? or medicine?))).ti,ab.	28,369
18	3 and 11 and 16	2,083
19	3 and 17	198
20	18 or 19	2,244
21	limit 20 to (conference abstract or conference paper or "conference review")	1,269
22	20 not 21	**989**

Execute search

CINAHL

#	Abfrage	Results (17th May 2022)
1	(MH "Readmission")	15,944
2	((TI Readmission# OR AB Readmission#) OR (TI Re-admission# OR AB Re-admission#) OR (TI Readmit? OR AB Readmit?) OR (TI Re-admit? OR AB Re-admit?) OR (TI Rehospitali? OR AB Rehospitali?) OR (TI Re-hospitali? OR AB Re-hospitali?))	19,492
3	S1 or S2	25,310
4	(MH "adverse drug event")	16,060
5	(MH "Medication Errors")	14,663
6	(((TI Drug# OR AB Drug#) OR (TI medication# OR AB medication#) OR (TI medicine# OR AB medicine#)) N3 ((TI related OR AB related) OR (TI associated OR AB associated)))	28,145
7	((TI Adverse OR AB Adverse) N1 ((TI drug OR AB drug) OR (TI medication OR AB medication) OR (TI medicine OR AB medicine)) N1 ((TI reaction# OR AB reaction#) OR (TI event# OR AB event#) OR (TI effect# OR AB effect#) OR (TI outcome# OR AB outcome#)))	9,508
8	((TI "side effect#" OR AB "side effect#") OR (TI "medication error#" OR AB "medication error#") OR (TI ADR# OR AB ADR#) OR (TI ADE# OR AB ADE#))	59,895
9	(((TI problem# OR AB problem#) OR (TI issue# OR AB issue#)) N3 ((TI drug# OR AB drug#) OR (TI medication# OR AB medication#)))	8,418
10	S4 OR S5 OR S6 OR S7 OR S8 OR S9	115,159
11	(MH "Risk Factors")	197,283
12	(MH "Risk Assessment")	130,087
13	((TI factor# OR AB factor#) OR (TI characteri? OR AB characteri?) OR (TI reason# OR AB reason#) OR (TI pattern# OR AB pattern#) OR (TI predictor# OR AB predictor#) OR (TI attribute# OR AB attribute#) OR (TI incident# OR AB incident#))	1,097,115
14	(((TI predict? OR AB predict?) OR (TI screening OR AB screening) OR (TI risk OR AB risk)) N2 ((TI model# OR AB model#) OR (TI tool# OR AB tool#) OR (TI score# OR AB score#) OR (TI assessment# OR AB assessment#)))	74,791
15	S11 OR S12 OR S13 OR S14	1,299,025
16	((((TI drug OR AB drug) OR (TI medication OR AB medication) OR (TI medicine OR AB medicine)) W1 ((TI group# OR AB group#) OR (TI class OR AB class) OR (TI classes OR AB classes))) OR (((TI causative OR AB causative) OR (TI causing OR AB causing)) W1 ((TI drug# OR AB drug#) OR (TI medication# OR AB medication#) OR (TI medicine# OR AB medicine#))))	5,626)
17	(MH "Readmission")	15,944
18	((TI Readmission# OR AB Readmission#) OR (TI Re-admission# OR AB Re-admission#) OR (TI Readmit? OR AB Readmit?) OR (TI Re-admit? OR AB Re-	19,492

	admit?) OR (TI Rehospitali? OR AB Rehospitali?) OR (TI Re-hospitali? OR AB Re-hospitali?))	
19	S17 OR S18	25,310
20	S3 and S10 and S15	259
21	S16 and S19	26
22	S20 or S21	**287**
