# Supplementary material for: Risk factors for medication-related short-term readmissions in adults – a scoping review
Source: BMC Health Serv Res. 2023 Sep 28;23:1037. doi: 10.1186/s12913-023-10028-2 (PMC10536731; doi:10.1186/s12913-023-10028-2)
Supplement: Supplementary file 2 — Additional file 2. [file 12913_2023_10028_MOESM2_ESM.pdf]

First Author<br>*Year*<br>*Country*	Study type<br>Study site<br>Study population<br>Causality (C)<br>Preventability (P)	Objectives<br>Readmission rate (RR)	Medication related risk factors /<br>Drug groups / medication related model variables
[1]<br>*Uitvlugt, EB*<br>*2021*<br>*The Netherlands*	Cross-sectional single-center observational study<br><br>Teaching hospital<br><br>1111 readmission<br><br>C: Yes → HARM<br>P: Yes → Schumock	Assess the prevalence and preventability of 30-day MRRs and description of risk factors, type of medication errors and types of medications involved in these MRRs<br><br>RR: 16% (181) were MRRs, thereof 40% (72) potentially preventable	<u>MRRs:</u><br><u>RF (preventable)</u>: Number of medication changes during index hospitalization (adjusted OR 1.14), having ≥3 hospitalizations 6 months before index hospitalization (adjusted OR 2.11).<br>Of the preventable medication-related readmissions, 35% were due to prescribing errors, 35% by non-adherence, and 30% by transition errors.<br><u>DG (preventable, top 5)</u>: diuretics [C03] (11, 15%), laxatives [A06A] (10, 14%), insulin [A10A] (7), oral antidiabetics [A10B] (4), antithrombotic agents [B01] (10%), medications for asthma/COPD [R03, R03DA04 (n=1)] (6, 8%), calcium antagonists [C08] (4), beta-blockers [C07] (3), medication affecting the RAAS [C09] (3), cardiac glycosides [C01A] (2), organic nitrates [C01DA] (1), alpha-blocker [C02CA] (1), corticosteroids [H02] (1), acetaminophen [N02BE01] (1), loperamide [A07DA03] (1), antiemetics [A04A] (1), opiates [N02A] (2), antiepileptics [N03A] (2), anti-Parkinson medication [N04] (1), mineral supplements [A12] (2), medication for treatment of hyperkalemia [V03AE] (1), antibiotics [J01] (2), infliximab [L04AB02] (1)<br><u>Medication errors (preventable)</u>: prescribing errors (25, 35% → underprescribing (19, 40%), dosage (6, 24%), inadequate monitoring (5, 20%), no indication (3, 12%)), across settings (22, 30% → transition errors (22, 30%)), medication-use (25, 35% → non-adherence (25, 35%))
[2]<br>*Cooper, JB*<br>*2020*<br>*USA*	Retrospective cohort study<br><br>Non-teaching community hospital<br><br>203 readmissions<br><br>C: Yes → Pellegrin<br>P: Yes → Clinical judgement	Determine the rate and type of MRPs attributable to 30-day readmissions after discharge from a general internal medicine department<br><br>RR: 50.2% (102) MRRs, thereof 57.8% (59) possibly preventable	<u>RF:</u> cancer, p=0.009 (others not statistically significant between readmitted due to MRP and not due to a MRP)<br><u>MRPs</u>:<br>Indication (34.3%), efficacy (19.6%), safety (18.6%), adherence (27.5%)<br><u>Indication</u> (17.2% (35), thereof 21 possibly preventable (35.6% of 59, 60% of 35)<br>• Medication not indicated (4.9% (10), thereof 4 possibly preventable (6.8% of 59, 40% of 10)<br>• Untreated condition (12.3% (25), thereof 17 possibly preventable (28.8% of 59, 68% of 25)<br><u>Efficacy</u> (9.9% (20), thereof 11 possibly preventable (18.6% of 59, 55% of 20)<br>• Ineffective drug (3.4% (7) thereof 2 possibly preventable (3.4% of 59, 42.9% of 7)<br>• Dose too low (6.4% (13), thereof 9 possibly preventable (15.3% of 59, 69.2% of 13)<br><u>Safety</u> (ADRs) (9.4% (19), thereof 14 possibly preventable (23.7% of 59, 73.7% of 19)<br>• ADRs due to inadequate monitoring (5.9% (12), thereof 9 possibly preventable (15.3% of 59, 75% of 12)<br>• ADRs due to contraindication (1.0% (2), thereof 2 possibly preventable (3.4% of 59, 100% of 2)<br>• ADRs due to dose too high (2.5% (5), thereof 3 possibly preventable (5.1% of 59, 60% of 5)<br><u>Adherence</u> (nonadherence) (13.8% (28), thereof 13 possibly preventable (22.0% of 59, 46.4% of 28)<br>• Nonadherence due to complexity (3.4% (7), thereof 4 possibly preventable (6.8% of 59, 57.1% of 7)<br>• Nonadherence due to administration (0.5% (1), thereof 1 possibly preventable (1% of 59, 100% of 1)<br>• Nonadherence due to access (drug cost) (3.4% (7), thereof 7 possibly preventable (11.9% of 59, 100% of 7)<br>• Nonadherence due to choice (6.4% (13), thereof 1 possibly preventable (1.7% of 59, 7.7% of 13)<br><u>Possibly preventable MRPs</u>:<br>Untreated condition (28.8% (17)), low dose (15.3% (9)), inadequate monitoring (15.3% (9)), lack of access to medications due to cost (11.9% (7))<br><u>DG of MRRs</u>:<br>• Cardiovascular agents [C] (45.1% (46, 33 potentially preventable)): antihypertensive (11.8%, 12, 10 potentially preventable) → lisinopril (3, 3 potentially preventable), antiplatelet (3.9%, 4, 2 potentially preventable) → aspirin (3, 2 potentially preventable), diuretic (13.7%, 14, 11 potentially preventable) → furosemide (11, 8

			potentially preventable), anticoagulant (8.8%, 9, 4 potentially preventable) → rivaroxaban (3, 2 potentially preventable), apixaban (3, 2 potentially preventable), warfarin (2, 0 potentially preventable)<br>• Endocrine agents [A] (7.8% (8), 3 potentially preventable): antidiabetic (5.9% (6, 2 potentially preventable)) → insulin (5, 1 potentially preventable)<br>• Anti-infective agents [J01] (10.8%, 11, 6 potentially preventable) → levofloxacin (2, 0 potentially preventable)<br>• Genitorurinary [G](4.9% (5, 2 potentially preventable))<br>• Neurology [N] (21.6% (22, 7 potentially preventable)): alcohol (10, 0 potentially preventable), amitriptyline (2, 2 potentially preventable)<br>• Pulmonary [R] (9.8% (10, 8 potentially preventable)) → Inhaled corticosteroids (6, 6 potentially preventable)<br>DG of possible preventable MRRs:<br>Diuretics [C03] (10.8% (11)), antihypertensives (9.8% (10)), inhaled therapies for COPD (7.8% (8))
[3] Whitaker, AS 2019 Australia	Retrospective cross-sectional study<br><br>General public hospital<br><br>196 readmissions<br><br>C: Yes → Clinician judgement<br>P: Yes → Clinician judgement	Investigation and categorization of medication-related 28-day representations to an emergency department.<br>Examination of demographic differences between medication-related and medication-unrelated emergency department representations.<br>Identification and categorization of MRPs associated with the medication-related representations<br><br>RR: 21% (41) MRR, thereof 59% (24) deemed preventable	DRP: toxicity or ADR (n=16, 50% preventable), undertreatment (n=15, 86.7% preventable) (condition undertreated (n=6), condition untreated (n=5), preventive therapy required (n=3), other untreated indication problem (n=1)), drug selection (n=3, 100% preventable) (wrong drug (n=2), no indication apparent (n=1)), over- or underdose (n=4, 100% preventable) (prescribed dose too high (n=1), prescribed dose too low (n=3)), compliance (n=6, 66.6% preventable) (under-use by consumer (n=2), erratic use of medication (n=3), difficulty using dosage form (n=1))<br>F: age (10.3 years older), number of medications (3.4 more medications)<br>DG: cardiovascular system (C) (n=14, preventable: n=11), anti-infectives for systemic use (J) (n=7, preventable: n=4), alimentary tract and metabolism (A) (n=5, preventable: n=5), nervous system (N) (n=5, preventable: n=3), respiratory system (R) (n=5, preventable: n=3), blood and blood-forming organs (B) (n=4, preventable: n=3), genitourinary system and sex hormones (G) (n=2, preventable: n=2), musculoskeletal system (M) (n=1, preventable: n=1)
[4] Dalleur, O 2021 USA	Retrospective observational cohort study<br><br>Academic hospital<br><br>10'275 discharges (LOS ≥ 1 day)<br><br>C: Yes → Naranjo<br>P: Yes → Schumock	Description of frequency, causes, severity and preventability of ADEs responsible for potentially avoidable 30-day readmissions in a medical population.<br>Qualitative assessment of which intervention could have potentially prevented the ADE readmissions.<br><br>RR: 13.1% MRRs<br>64.3% (MRP main cause)<br>35.7% (MRP contributing cause).<br>92.9% of all MRRs which were deemed possible preventable beforehand (only those analyzed) were judged to be preventable using the Schumock statements.	Causes: Inappropriate prescribing (48.6%)<br>Overprescribing ("no indication") (10%):<br>Analgesics [N02]→ Altered mental status (4%); Diuretics [C03] →Dehydration (3%); Antithrombotic agents [B01] → Gastrointestinal bleeding (1%); Combination of one antihypertensive [C02, C07, C09], one agent acting on the renin-angiotensin system [C09] and one vasodilator [C01D] (3 drugs)→ Hypotension (1%)<br>Misprescribing ("indication, but suboptimal drug prescribing") (27%):<br>Diuretics [C03], beta-blocking agents [C07] and/or agents acting on the renin-angiotensin system [C09] (overdosage, drug-drug interactions) → Hypotension, dehydration, and/or acute renal failure (11%); Antithrombotic agent [B01] (overdosed or drug-drug interactions) → Bleeding (4%); Insulin [A10] (overdosage)→ Hypoglycemia (4%); Opioids and drugs used against addiction [N02A] (overdosage) → Altered mental status (3%); Corticosteroid for systemic use [H02] (overduration)→ Heart failure (1%); Antibacterial for systemic use [J01] (underdosage)→ Recurrent infection (1%); Antiprotozoal (chloroquine) [P01; P01BA01] (drug-disease interaction) → Prolonged QT interval (1%)<br>Underprescribing ("indication, but no drug") (11%):<br>Analgesics [N02] → Uncontrolled pain (4%); Diuretics [C03] → Volume overload and heart failure (3%); Drugs against constipation [A06A] (lactulose to prevent hepatic encephalopathy) → Liver failure (1%); Cardiac therapy (antiarrhythmic) [C01B] → Tachycardia (1%); Antibacterial for systemic use [J01] → Recurrent infection (1%)<br>Other: including suboptimal patient monitoring/education (51.4%) [Adherence issues (8.6%), lack of monitoring (44.3%)]:<br>Antineoplastic [L01] →Neutropenia and fever (9%), Gastro-intestinal symptoms (6%), Acute kidney injury (1%), Heart failure (1%), Liver failure (1%); Antibacterial for systemic use [J01] (recent switch to another agent) →

			Allergic reaction (1%), Recurrent infection (4%), Seizure (1%); Immunosuppressant agent [L04] → Acute kidney injury (3%), Neutropenia and fever (1%); Antithrombotic agent [B01] → Bleeding (4%); Diuretics [C03]→ Allergy (1%), Volume overload and heart failure (3%); Analgesics [N02] → Constipation (1%), Altered mental status (1%), Liver failure (1%); Insulin [A10]→ Hypoglycemia (3%); Immunoglobulin [J06B]→ Fever (1%); Cardiac therapy (antiarrhythmic) [C01B] → Hypothyroidism (1%); Corticosteroid for systemic use [H02] → Weakness (1%); Trial medication → Gastrointestinal symptoms (1%)<br>Top 10 DG: Antineoplastic agents [L01] (18.6%), diuretics [C03] (14.3%), analgesics [N02] (12.9%), antithrombotic agents [B01] (10.0%), systemic antibacterials [J01](8.6%), antidiabetics [A10] (7.1%), beta-blocking agents [C07] (4.3%), immunosuppressant agents [L04] (4.3%), cardiac therapy [C01] (2.9%), agents acting on renin-angiotensin system [C09] (2.9%)
[5]<br>Dreyer, R<br>2019<br>South Africa	Retrospective descriptive analysis<br><br>Academic hospital<br><br>11'826 discharges<br><br>C: No<br>P: Yes → Clinician judgement	Analysis of 30-day all-cause readmissions of patients discharged from an internal medicine department.<br>Comparison of characteristics between avoidable and unavoidable readmissions and subsequent identification of potentially preventable factors.<br>Analysis of the LACE and mLACE index for its effectiveness to detect readmissions in the studied health system<br><br>RR: 10.5% (1242)<br>Due to ADR: 33 (2.6% of readmissions), thereof 19 deemed preventable<br>Due to non-compliance: 10% of all readmissions	ADRs → DG:<br>Warfarin toxicity [B01AA03] (17/33)<br>DRPs:<br>Non-compliance (deemed unavoidable)
[6]<br>Toh, MR<br>2014<br>Singapore	Retrospective cross-sectional study<br><br>National electronic database (discharges from all public, tertiary hospitals)<br><br>649 readmissions<br><br>C: Yes → All readmissions were reviewed for MRPs. Considered only if it was stated in the case notes to be caused by a MRP (physician judgment)<br>P: No	Analysis of the association between number of medication doses per day and number of 15-day readmissions and association between readmission frequency with other medical and sociodemographic variables<br><br>RR: 15.9% MRR (103)	MRPs:<br>Non-compliance (39), overdose (10), hypoglycemia (7), dehydration (7), hypotension (5), giddiness, liver function test abnormalities (5), hyponatremia (4), hypokalemia (4), nausea / vomiting / diarrhea (4), thrombocytopenia (4), rashes (4), anemia (2), metabolic alkalosis (1), constipation (1), underdose (1)<br>All-cause:<br>RF: number of doses per day, number of medications
[7]<br>Willson, MN<br>2014<br>USA	Retrospective parallel-group case-control design<br><br>4 acute care hospitals	Comparison of 30-day readmission and association with MRCI in patients readmitted due to an ADE and patients readmitted with a non-ADE related cause.	RF:<br>• Higher overall MRCI score at admissions and discharge (best predictor: ≥8)<br>• Every section was associated with a higher ADE-related readmission. (section A: dosage form, section B: dosing frequency, section C: additional directions required for administration)

	92 patients readmitted due to an ADE and matched controls (same disease classification)<br><br>C: Yes → Coded with an adverse event as accidental poisoning<br>P: No	Determination if MRCI could predict readmissions.<br><br>RR: 92 (due to ADE), preselected and matched to controls	• Number of medications
[8] Banholzer, S 2021 Switzerland	Retrospective study<br><br>University hospital<br><br>4792 readmissions<br><br>C: Yes → Clinician judgement<br>P: No	Characterization of ADRs leading to 30-day representation to the ED department. Assessment of the reporting frequency of such ADRs to the Swiss national pharmacovigilance center.<br><br>RR: 27% (1294) ED-readmissions and thereof, 20.9% (270) cases ADR-related ED readmission	DG: antineoplastic/immunomodulation agents (35%), antithrombotic agents (25%), nervous system drugs (16%); chemical subgroups: glucocorticoids (11%), platelet aggregation inhibitors (9%), heparins (8%), vitamin K antagonists (7%), calcineurin inhibitors (5%) and other immunosuppresants such as mycophenolate or mTOR inhibitors (4%). Sometimes it was caused by the drug alone, through DDI or by the drug itself but through a DDI (e.g. increased toxicity).<br>ALIMENTARY TRACT AND METABOLISM (A) (n=6; 1.3% → Proton pump inhibitors (A02BC; n=1: 0.2%), Insulins and analogues for injection, fast-acting (A10AB; n=2: 0.4%), Insulins and analogues for injection, long-acting (A10AE; n=1: 0.2%), Biguanides (A10BA; n=1: 0.2%), Calcium, combinations with vitamin D and/or other drugs (A12AX; n=1: 0.2%))<br>BLOOD AND BLOOD FORMING ORGANS (B) (n=118; 24.7% → Vitamin K antagonists (B01AA; n=35: 7.3%), Platelet aggregation inhibitors excl. heparin (B01AC; n=45: 9.4%), Heparin group (B01AB; n=36: 7.5%), Iron bivalent, oral preparations (B03AA; n=1: 0.2%), Other antithrombotic agents (B01AX: n=1: 0.2%))<br>CARDIOVASCULAR SYSTEM (C) (n=24; 5.0% → Thiazides, plain (C03AA; n=1: 0.2%), Sulfonamides, plain (C03BA n=9: 1.9%), Low-ceiling diuretics and potassium-sparing agents (C03EA; n=1: 0.2%), HMG-CoA reductase inhibitors (C10AA; n=1: 0.2%), Dihydropyridine derivates (C08CA; n=1: 0.2%), Beta blocking agents, selective, and other diuretics (C07CB; n=1: 0.2%), Beta blocking agents, selective (C07AB; n=2: 0.4%), Angiotensin-II receptor blockers (ARBs) and diuretics (C09DA; n=1: 0.2%), Angiotensin-II receptor blockers (ARBs), plain (C09CA; n=2: 0.4%), Aldosterone antagonists (C03DA; n=3: 0.6%), ACE inhibitors and diuretics (C09BA; n=1: 0.2%); ACE inhibitors, plain (C09AA; n=1; 0.2%))<br>GENITO URINARY SYSTEM AND SEX HORMONES (G) (n=2; 0.4% → Imidazole derivates (G01AF; n=1: 0.2%), Drugs for urinary frequency and incontinence (G04BD; n=1: 0.2%))<br>SYSTEMIC HORMONAL PREPARATIONS, EXCL. SEX HORMONES AND INSULINS (H) (n=50; 10.5% → Glucocorticoids (H02AB; n=50: 10.5%))<br>ANTIINFECTIVES FOR SYSTEMIC USE (J) (n=26; 5.5% → Carbapenems (J01DH; n=3: 0.6%), 3rd Generation cephalosporins (J01DD; n=2: 0.4%), 4th Generation cephalosporins (J01DE; n=1: 0.2%), Fluorquinolones (J01MA; n=3: 0.6%), Combinations of penicillins, incl. beta-lactamase inhibitors (J01CR; n=10: 2.1%), Lincosamides (J01FF; n=2: 0.4%), Macrolides (J01FA; n=3: 0.6%), Penicillins with extended spectrum (J01CA; n=1: 0.2%), Protease inhibitors (J05AE; n=1: 0.2%))<br>ANTINEOPLASTIC AND IMMUNOMODULATING AGENTS (L) (n=169; 35.4% → Other alkylating agents (L01AX n=3: 0.6%), Other antineoplastic agents (L01XX; n=1: 0.2%), Other immunosuppressants (L04AX; n=5: 1.0%), Other cytotoxic antibiotics (L01DC; n=1: 0.2%), Anthracyclines and related substances (L01DB; n=14: 2.9%), Calcineurin inhibitors (L04AD; n=22: 4.6%), Folic acid analogues (L01BA; n=1: 0.2%), Interferons (L03AB; n=1: 0.2%), Colony stimulating factors (L03AA; n=1: 0.2%), Methylhydrazines (L01XB; n=1: 0.2%), Monoclonal antibodies (L01XC; n=16: 3.4%), Platinum compounds (L01XA; n=16: 3.4%), Podophyllotoxin derivates (L01CB; n=11: 2.3%), Protein kinase inhibitors (L01XE; n=3. 0.6%), Purine analogues(L01BB; n=1: 0.2%), Pyrimidine analogues (L01BC; n=14: 2.9%), Selective immunosuppressants (L04AA; n=20: 4.2%), Nitrogen mustard analogues (L01AA; n=18: 3.8%),

			Taxanes(L01CD; n=4: 0.8%), Vinca alkaloids and analogues (L01CA; n=16: 3.4%))<br>MUSCULO-SKELETAL SYSTEM (M) (n= 6; 1.3% → Propionic acid derivatives (M01AE; n=1: 0.2%), Other centrally acting agents (M03BX; n=2: 0.4%), Preparations inhibiting uric acid production (M04AA; n=1: 0.2%), Bisphosphonates (M05BA; n=2: 0.4)<br>NERVOUS SYSTEM (N) (n=74; 15.5% → Other general anesthetics (N01AX; n=1: 0.2%), Other antidepressants (N06AX, n=8: 1.7%), Other antiepileptics (N03AX; n=3: 0.6%), Other opioids (N02AX, n=2: 0.4%), Anilides (N02BE; n=1: 0.2%), Benzodiazepine derivatives (N05BA; n=6: 1.3%), Benzodiazepine related drugs (N05CF, n=2; 0.4%), Carboxamide derivatives (N03AF; n=4: 0.8%), Diazepines, oxazepines, thiazepines and oxepines (N05AH; n=2: 0.4%), Dopamine agonists (N04BC; n=3: 0.6%), Dopa and dopa derivatives (N04BA; n=1: 0.2%), Fatty acid derivatives (N03AG; n=2: 0.4%), Hydantoin derivatives (N03AB; n=1: 0.2%), Drugs used in nicotine dependence (N07BA; n=1: 0.2%), Drugs used in opioid dependence (N07BC; n=4: 0.8%), Natural opium alkaloids (N02AA; n=6: 1.3%), Non-selective monoamine reuptake inhibitors (N06AA; n=1: 0.2%), Phenylpiperidine derivatives (N02AB; n=11: 2.3%), Pyrazolones (N02BB; n=4: 0.8%), Salicylic acid and derivatives (N02BA; n=1: 0.2%), Selective serotonin reuptake inhibitors (N06AB; n=10: 2.1%))<br>RESPIRATORY SYSTEM (R) (n=1; 0.2% → Anticholinergics (R03BB; n=1: 0.2%))<br>VARIOUS (V) (n=1; 0.2% → Detoxifying agents for antineoplastic treatment (V03AF; n=1: 0.2%))<br>RF: Readmitted patients with an ADR were older (p<0.001) than readmitted patients without ADRs (probably due to polypharmacy, impaired renal function or other comorbidities). Most readmitted patients were returned home after discharge.
[9]<br>*Nikolaus, T*<br>*1992*<br>*Germany*	Retrospective cohort study<br><br>Geriatric hospital<br><br>67 geriatric patients repeatedly readmitted (331 readmissions)<br><br>C: Yes → Clinician judgement<br>P: Yes → Clinician judgement	Analysis of causes of frequently readmitted geriatric patients, to assess preventable causes and to formulate preventive measures.<br>Readmission time period was set to 3 weeks.<br><br>RR: 26.3% (87) of the 331 readmissions were during the first 3 weeks after last discharge	DRPs:<br>• TOC problem: 14 cases of insufficient medication and/or family doctor supply during weekend. Most of them had a heart failure diagnosis (55.5% deemed potentially preventable)<br>• 13.8% (12): Non-compliance with medications or diet (58.3% deemed potentially preventable)<br>• 11.5% (10): ADRs as cause of readmission, thereof 5/10 due to hypoglycemia caused by sulfonylureas [A10BB] (40% deemed potentially preventable)
[10]<br>*Ekerstad, N*<br>*2017*<br>*Sweden*	Prospective observational study<br><br>Hospital group: 5 hospitals<br><br>390 patients with frailty (FRESH criteria) and ≥75 years<br><br>C: Yes → Naranjo<br>P: Yes → Hallas and underuse of evidence-based treatment	Study the causes of 30-day readmissions of frail elderly patients, particularly in the context of medications, including both over- and underuse of evidence-based treatments.<br><br>RR: 24.6% (96). Thereof, 13.5% (13) due to ADR and thereof 2.1% (2) preventable<br>Underuse of evidence-based treatment (n=19)	All-cause:<br>RF: Polypharmacy (≥10 drugs) (62.5% vs. 54%, p<0.001)<br>ADR-caused<br>Probably avoidable: digoxin/digitalis glycoside [C01AA05] → Cause: nausea/tiredness and falling<br>Not avoidable: oxycodone/opioid [N02AA05] (constipation), citalopram/antidepressant [N06AB04] (constipation), carbamazepine/antiepileptic [N03AF01] (falling), metoprolol/beta-blocker [C07AB02] (fainting → orthostatism), duloxetine/antidepressant [N06AX21] (falling), warfarin/anticoagulant [B01AA03] (gastrointestinal bleeding), acetyl salicylic acid/salicylate [B01AC06] and clopidogrel/P2Y12 inhibitor [B01AC04] (gastrointestinal bleeding → enterocolitis), metoprolol/beta-blocker [C07AB02] and felodipine/calcium channel blocker [C08CA02] (vertigo, palpitations → supraventricular tachycardia), clindamycin/macrolide [J01FF01] (fever, diarrhoea → enterocolitis), warfarin/anticoagulant [B01AA03] (abdominal pain → muscle haemorrhage), furosemide/loopdiuretic [C03CA01] and spironolactone/potassium-sparing diuretic [C03DA01] (tiredness → urinary tract infection)<br>Underuse of evidence-based drug (n = 19): Avoidable: heart failure (n = 11 → anticoagulant [B01AA, B01AB, B01AF, B01AX] (1), acetyl salicylic acid [B01AC06] (1), beta-blocker [C07A] (6), ACE/ARB [C09] (6)), atrial fibrillation (n = 3, anticoagulants [B01AA, B01AB, B01AF, B01AX] (3)), pneumonia (n = 1, antibiotics [J01]), COPD

			(n = 1, antibiotics [J01]), enterocolitis (n = 1, antibiotics [J01]), unstable angina (n = 1, dual antiplatelet therapy [B01AC])
[11]<br>*Rothwell, M*<br>*2011*<br>*Australia*	Retrospective case-note review<br><br>1 regional and 2 rural hospitals<br><br>Patients ≥65 years and ≥3 medications<br><br>C: Yes → Clinician judgment<br>P: Yes → Clinician judgment	Identification of unplanned 28-readmissions attributable to medication and to assess preventability.<br>In the MRRs, identification of the medication management information supplied to the GP on initial discharge and if these patients were seen by a pharmacist during initial hospital stay and readmission.<br><br>RR: 23% (39) of all readmissions were MRRs, thereof 87% (34) preventable	DRPs: Inadequate communication/monitoring (41%) and inappropriate/suboptimal pharmacological therapy at discharge (62%).<br>DG (preventable): ACE inhibitor [C09AA] (n=3 → combined with diuretic [C03] leading to acute renal failure (inadequate monitoring); continued to take AT-II receptor antagonist [C09AC] leading to acute renal failure (inadequate monitoring), ceased on admission and readmitted with hypertension (suboptimal therapy)), calcium channel blocker [C08] (n=2) → adverse medication event not identified during initial admission (suboptimal therapy), symptoms arising from medication ceased during the initial admission (suboptimal therapy)), beta-blocker [C07] (n=3 → medication decreased and subsequent atrial fibrillation not identified during the initial admission (suboptimal therapy), medication increased during initial admission and not documented on the discharge summary (inadequate monitoring/communication), fall due to non-adherence with medication for atrial fibrillation (inadequate monitoring), diuretic [C03] (n=7, hyponatraemia (inadequate monitoring/communication), heart failure due to non-adherence (inadequate monitoring/communication), heart failure due to inadequate medication and education (inadequate monitoring/communication), heart failure due to decrease in medication (suboptimal therapy), heart failure due to not starting medication (suboptimal therapy), medication-induced syncope (suboptimal therapy)), antiplatelet [B01AC] (n=2, medication-induced GI bleed not picked up on initial admission (suboptimal therapy), aspirin [B01AC06] and clopidogrel [B01AC04] ceased due to GI bleed an patient was readmitted with chest pain (suboptimal therapy)), peripheral vasodilator [C04] (n=1 → adverse drug event not identified on initial admission (suboptimal therapy)), antibiotic [J01] (n=2 → non-compliance (same as for initial admission) (inadequate monitoring/communication), antibiotic-induced clostridium difficile (suboptimal therapy)), insulin [A10A] (n=2, fall resulting from hypoglycaemia (inadequate monitoring/com communication), ketoacidosis from non-compliance due to initial illness (suboptimal therapy)), proton pump inhibitor [A02BC] (n=3, inadequate therapy for gastro-oesophageal reflux disease not picked up during the initial admissions (suboptimal therapy), medication-induced GI bleed (suboptimal therapy)), iron [B03A] (n=3 → constipation not picked up during initial admission (suboptimal therapy)), temazepam [N05CD07] (n=1 → medication ceased on initial admission but patient continued to take it leading to a fall (inadequate monitoring/communication)), warfarin [B01AA03] (n=2 → bled into shoulder (INR 4.7) (inadequate monitoring/communication, INR>10 (suboptimal therapy)), heparin [B01AB01] (n=1 → post-surgical deep vein thrombosis (suboptimal therapy)), analgesic [N02] (n=3 → inadequate pain relief (suboptimal therapy)), hydroxyurea [L01XX05] (n=1 → myelosuppression (inadequate monitoring/communication)), donepezil [N06DA02] (n=1, adverse drug event not picked up during initial admission (suboptimal therapy))
[12]<br>*Weir, DL*<br>*2020*<br>*Canada*	Prospective cohort study<br><br>University hospital<br><br>2402 discharges of patients ≥65 years and with ≥1 medication(s)<br><br>C: Yes → Leape-Bates<br>P: No	Estimation of the number of PIMs prescribed to patients at hospital discharge and to analyze their risk of adverse events 30 days after discharge, including readmissions and ED visits<br><br>RR: 850 (35.4%) (all-cause)	All-cause<br>RF: New PIM and community-continued PIMs
[13]<br>*Glans, M*<br>*2021*<br>*Sweden*	Retrospective medical record study<br><br>255 bed hospital	Find risk factors associated with 30-day MRRs in patients ≥65 years.	Significant patient characteristics: older age, more comorbidities, more often dependent on help from municipality caregivers, more use of regular drug, more often subject to dosage adjustment, and using a FRID (fall-risk increasing drug) (comparison of MRRs and non-readmitted patients)<br>RF: Charlson Comorbidity Index (OR 1.15), excessive polypharmacy (≥10 regular medications) (OR 1.74), having

	360 readmitted and 360 non-readmitted patients; ≥65 years<br><br>C: Yes → AT-HARM 10<br>P: No	Focuses are on living arrangement, polypharmacy, PIMs, medication regimen changes.<br><br>RR: 40% (143) of all readmissions were MRR	adjustments in medications at primary discharge (OR 1.63) and living in own home, alone compared to with someone (with spouse: OR 0.59 or in nursing home OR 0.45), initial admission to emergency department (OR 5.13), number of admissions in past 12 month (OR 1.33)<br>Preventive factors: new medication started in index admission (OR 0.54)<br>Categorization of MRP: ADRs (n = 97, 49%), laboratory results/vital signs causing the readmissions (e.g. hypokalemia due to diuretics) (n = 37, 19%), interaction or contraindication (n = 8, 4%), undertreatment/underprescribing (n = 46, 23%), handling problem (e.g. incorrect use of inhaler) (n = 1, 1%), cessation or withdrawal of therapy (n = 7, 4%)
[14]<br>Barnett, NL<br>2017<br>UK	Retrospective cohort study<br><br>658-bed district general hospital<br><br>744 patients at risk for readmission<br><br>C: Yes<br>P: Yes	Description of the effect of an integrated medicines management service on 30-day preventable MRRs.<br><br>RR: 16% (119) readmitted of 744 patients receiving the service, thereof 1.7% (2) a preventable MRR;<br>no analysis / validation of the PREVENT tool published	PREVENT model variables:<br>• Physical impairment: For example difficulties with swallowing, impaired dexterity, poor vision, hard of hearing, poor mobility<br>• Frailty: identified as frail using accepted methods<br>• Adherence issues / compliance support<br>• Cognitive impairment: Patient is unable to take medication regularly without support as they have a condition which affects their memory, e.g. delirium, dementia<br>• New diagnosis/exacerbation of disease: Admission is related to poor management of medication for a long term condition or deterioration of organ system function, e.g. renal cardiac. Previous admission or A&E attendance within 30 days. Depression, high level of stress, other mental health, alcohol or drug abuse<br>• Medicines related admission / risk from specific medicines: Patient is taking a high risk medicine (e.g. anticoagulants/antiplatelets, insulin, oral hypoglycemics, NSAIDs. benzodiazepine, antihypertensives, diuretics, beta-blocking agents, opioids, methotrexate, injectable medicines, drugs requiring therapeutic drug monitoring esp. with no monitoring) which the patient is unable to manage. Patient has a complex of medicine regimen, recent stop, start or change in medicines or Polypharmacy which the patient is unable to manage<br>• Cultural/social: Patient cannot manage daily activities independently or has carers to help with daily activities but not medicines. Patient has cultural beliefs around illness and treatment impacting medication adherence. Patient has social issues such as no ficed abode, unkempt etc. which o,pacts them taking medications, smoker
[15]<br>Koekkoek, D<br>2011<br>USA	Retrospective cohort study<br><br>4 community hospitals<br><br>300 readmissions<br><br>C: No<br>P: Yes	Examination of 21-day readmission and association with preventability from hospital medicine experts.<br>Through chart review, patient factors and care processes that affect preventability were identified.<br>Description of clinicians' ideas for preventing future readmissions<br><br>RR: NA	RF of preventable readmission: >3 new medications, having anticoagulation treatment<br>Intervention that might have prevented readmissions related to medications: different discharge medications/dosage, improve medication reconciliation or education
[16]<br>Classen, DC<br>2010<br>USA, Puerto Rico, Virgin Islands	Retrospective cohort study<br><br>National surveillance system<br><br>Medicare population: 25'145 discharges	Identify exposure to warfarin, heparin, low molecular weight heparin/factor Xa inhibitors, insulin/oral hypoglycemic agents, digoxin, and systemic antibiotics and association with adverse events, including 30-day readmissions	Drug groups:<br>Patients with ADEs associated with low molecular weight heparin [B01AB]/factor Xa inhibitors [B01AF] had significantly higher readmission rates.

	C: No<br>P: No	RR: NA	
[17]<br>*Porter, M*<br>*2019*<br>*USA*	[Retrospective cohort study]<br><br>Academic medical center<br><br>2621 patients<br><br>C: No<br>P: No	Examination of characteristics of patients with a high and a low 30-day readmission rate and those never readmitted over a 3-year period<br><br>RR: 897 (34.4%):<br>1 readmission<br>314 (12.0%):<br>2 or more readmissions	RF: Number of medications<br>→ Mean numbers: 8.7 (non-readmitted) vs. 9.8 (readmitted one time), 14.1 (readmitted more than one time) → p-values: 0.0052 (non-readmitted vs. readmitted one time), <0.001 (readmitted one time vs. readmitted more than 1 time and non-readmitted vs. readmitted more than 1 time)<br>→ More than 12 medications: OR 1.380, p=0.022 to be readmitted more than one time
[18]<br>*Wetherell, M*<br>*2017*<br>*USA*	Prospective interview<br><br>Academic medical center<br><br>117 medicine patients<br><br>C: No<br>P: No	Ask clinical teams about patients and if they would be surprised if these would be readmitted within 30 days.<br>Ask clinical teams to identify patients most likely to be readmitted.<br><br>RR: 10.7%	All-cause<br>RF: Number of discharge medications (14.2 vs. 10.8, p=0.032, OR 1.07)
[19]<br>*Balla, U*<br>*2008*<br>*Israel*	Prospective case-control study<br><br>Academic medical center<br><br>1916 discharges from medical departments<br><br>C: No<br>P: Yes → Clinician judgement	Assessment of preventability of 30-day readmissions and the ability to use readmissions as a tool to expose, address, and improve quality of care problems.<br><br>RR: 14.1% (271)	RF:<br>• Number of medications (all readmissions 6.4 vs. matched controls 5.1 (p<0.001); preventable vs. non-preventable not statistically significant)<br>MRPs (deemed preventable):<br>• Suboptimal drug treatment (important treatment withheld or given suboptimally): 44%
[20]<br>*Aljishi, M*<br>*2014*<br>*New Zealand*	Retrospective case control study<br><br>Academic hospital<br><br>3964 patients admitted to a general medicine department<br><br>C: No<br>P: No	Investigation 30-day readmissions to the same general medicine department and identification of risk factors and association with mortality<br><br>RR: 4.97% (197)	RF:<br>Number of medications (7.7 vs. 6.0; p=0.001) → OR 1.09, p=0.02
[21]<br>*Schwab, C*<br>*2018*<br>*France*	Retrospective, matched, case-control study<br><br>University hospital<br><br>6574 discharges of patients ≥75 years	Identification of iatrogenic risk factors for 30-day readmissions.<br>Evaluate the rate of MRRs among all readmissions and compare it to the readmissions rate for other reasons.<br><br>RR: 6.4% (422)	All-cause:<br>RF:<br>• Number of drugs (p=0.002), polypharmacy (>5 drugs) (p=0.013), PIMs (p=0.006)[Beers, probably], systemic hormonal preparations [H] and nervous system drugs [N] (p<0.001)<br>DRRs:<br>DRPs:<br>• Overdose of antihypertensives [C02, C07, C09] (30%) → fall (6.4%) and hypotension (8.5%)<br>RF:

	C: Yes<br>P: (Yes) → Deemed MRRs as mostly avoidable, no separate analysis	11.4% (48) of readmitted patients were MRRs	• Number of drugs (p<0.001)<br>• Number of PIMs (p=0.008) [Beers, probably]
[22]<br>*Beckner, A*<br>*2021*<br>*USA*	Mixed method study<br><br>All patients enrolled in a specific home health agency and discharged from hospital and readmitted to an affiliated with a local hospital<br><br>126 readmitted patients needing home health services<br><br>C: No<br>P: No	Explore the relationship between medication nonadherence, 30-day readmission, and factors contributing to medication non-adherence in patients needing home health services<br><br>RR: NA	RF: Insufficient communication between caregivers regarding medications, no prescription for new medications obtained, pharmacy wrongdoing (issues with medication supply), non-adherence due to lack of education, non-adherence due to pill hoarding (cost, intention)
[23]<br>*Lau, MHM*<br>*2017*<br>*China*	Retrospective single-center chart review study<br><br>Public hospital<br><br>165 patients ≥75 years and ≥5 medications and discharged with one or more of the following medications: verapamil/diltiazem, tricyclic antidepressants, chlorpromazine, clozapine, flupenthixol, zuclopenthixol, antipsychotics except quetiapine, anticholinergics, antimuscarinics, prochlorperazine, metoclopramide, anti-muscarinic bronchodilators, non-selective beta-blocker, COX-2 selective NSAID, oral bisphosphonate, thiazolidinedione, oestrogens<br><br>C: No<br>P. No	28-day emergency readmission and its association with PIMs defined by selected STOPP version 2 criteria related to drug-disease interactions<br><br>RR: 42 (25.5%)	RF:<br>• PIMs defined by STOPP version 2: OR 6.476, p<0.001<br>• Anticholinergics [N04A, R03BB, S01FA, A03AA, A03AB]: OR 12.667, p<0.001<br>• PIMs related to gastrointestinal disorder: OR 5.405, p=0.025<br>• PIMs related to gout: OR 3.490, p=0.004
[24]<br>*Pavon, JM*<br>*2014*<br>*USA*	Retrospective observational study	Use of electronic health record data to examine the association between exposure to individual medication classes during an initial admission and 30-day readmission.	All-cause:<br>DG: anticonvulsants [N03A] (OR 1.26, 1.08-1.48), benzodiazepines [N05BA] (OR 1.23, 1.04-1.44), corticosteroids [H02] (OR 1.26, 1.07-1.50), opioids [N02A] (OR 1.25, 1.06-1.47), antidepressants [N06A] in cardiology service (OR 1.85, 1.16-2.96), opioids [N02A] in medicine service (OR 1.94, 1.17-3.22) (greater odds than in surgery)

	Tertiary and quaternary academic health system<br><br>4627 discharges of patients ≥60 years<br><br>C: No<br>P: No	Examination of these associations differed according to inpatient service type.<br><br>RR: 21% (955)	
[25]<br>*Ibarra Mira, M.L*<br>*2021*<br>*Spain*	Prospective controlled, quasi-experimental study<br><br>120-bed tertiary hospital<br><br>163 polymedicated (>5 medications) patients at high risk for potentially avoidable readmissions according to the HOSPITAL score >7<br><br>C: No<br>P: No	Determination if a pharmaceutical care discharge program in polymedicated patient at high risk of potentially avoidable readmissions according to the HOSPITAL score improves 30-day readmission rates.<br><br>RR: 18.4% intervention group<br>25.6% in control	<u>All-cause:</u><br><u>RF (</u>multivariate analysis): Number of medications (OR 1.07, 1.01-1.14)<br>(HOSPITAL score elsewhere published, no medication-related factors)
[26]<br>*Pereira, F*<br>*2021*<br>*Switzerland*	Retrospective registry-based cohort study<br><br>Public general hospital<br><br>20’422 patients with ≥5 medications and ≥65 years<br><br>C: No<br>P: No	Use of hospital register data to prioritize risk factors for 30-day hospital readmission in older adults<br><br>RR: 7.8%	<u>RF:</u> Number of drugs<br><u>DG:</u> Blood and blood-forming organs [B] (OR 1.089), systemic hormonal preparations, excluding sex hormones and insulins [H] (OR 1.207), respiratory system drugs [R] (OR 1.146), drugs for functional gastrointestinal disorders [A03] (OR 1.424), antiemetics and antinauseants [A04] (OR 3.216), drugs for constipation [A06] (OR 1.195), drugs used in diabetes [A10] (OR 1.125), vitamins [A11] (OR 1.201), antihypertensives [C02] (OR 1.771), diuretics [C03] (OR 1.149), beta-blocking agents [C07] (OR 1.156), psycholeptics [N05] (OR 1.130)<br><u>Interactions</u>: Beta-blocking agents and drugs for acid-related disorders [C07 and A02] (OR 1.367)
[27]<br>*Basnet, S*<br>*2018*<br>*USA*	Retrospective multicenter cohort study<br><br>Large health system including among others two large tertiary academic hospitals<br><br>25’190 patients ≥65 years<br><br>C: N<br>P: N	Determination of the relationship between increasing medication use and 30-day readmission in patients ≥65 years. Exploration between risk factors, including comorbidity, and 30-day readmission.<br><br>RR: 16%	<u>RF:</u> Number of medications (p<0.001) → 1-5 medications → 12.3%, 21-30 medications → 27.1% → Multivariate: Each medication → 4% increase. Total medication: OR 1.04, p>0.001<br>Number of Beers also associated in univariate analysis but when controlled for number of medications → no association
[28]<br>*Lohman, MC*<br>*2017*<br>*USA*	Cross-sectional analysis<br><br>132 home health agencies	Describe the prevalence and types of PIMs (using Beers criteria) at start of home health care among patients receiving home health care service.	<u>RF:</u> Any PIM (excluding NSAIDs) → aOR: 1.13 (1.07-1.19), number of PIMS 2+, AOR: 1.14 (1.03-1.25), number of PIMs (excluding NSAIDs) 1+ aOR: 1.10 (1.04-1.17), 2+ aOR: 1.27 (1.14-1.41) → Beers<br><u>PIM DG</u>: Benzodiazepines aOR: 1.13 (1.06-1.21), cardiovascular (Guanabenz, guanfacine, methyldopa, disopyramide, digoxin (>0.125 mg/day), nifedipine) aOR: 1.19 (1.07-1.33), anti-infective (nitrofurantoin), aOR:

	35’638 home health care nursing patients ≥65 years<br><br>C: No<br>P: No	Estimate the association between PIM use at start of home health care and subsequent 30-day hospitalization, including rehospitalization.<br><br>RR: 14.6% (5205)	1.48 (1.06-2.05), gastrointestinal (metoclopramide (except with diagnosis of gastroparesis), oral mineral oil, trimethobenzamide), aOR: 1.33 (1.05-1.68)
[29]<br>*Sorensen, A*<br>*2021*<br>*USA*	Retrospective cohort study<br><br>Academic medical center<br><br>494 patients ≥65 years receiving the intervention and a matched control group of patients with similar demographic and clinical characteristics receiving usual care<br><br>C: No<br>P: No	Evaluation of a care transition intervention that aimed at improving medication management and medication safety and association with 30-day readmission<br>Conduction of a root cause analysis to find risk factors for 30-day readmission; not elsewhere published, results of analysis not published<br><br>RR: 10.6% in intervention group, 21.4% in control group	RF: ≥8 prescription medications (others from root-cause analysis not medication-related)
[30]<br>*Fung, L*<br>*2020*<br>*USA*	Retrospective chart review<br><br>Academic medical center<br><br>4482 discharged patients<br><br>C: No<br>P: No	Validation of the UCSD-Rx risk score by assessing its ability to predict 30-day readmissions and medication errors at discharge<br><br>RR: 13%	Model-variables: (C-statistics: 0.66) (only medication-related)<br>Home medication list not reviewed during admission, polypharmacy ≥8 medications, discharge medication list changed and not printed out, inpatient order for high-risk medication (anticonvulsant, antidiabetic, antiretroviral, transplant medication, anticoagulant, antiplatelet, long-acting opioid), outpatient order for high-risk medications (anticonvulsant, antidiabetic, antiretroviral, transplant medication, anticoagulant, antiplatelet, long-acting opioid, IV medication), enoxaparin on the outpatient medication list
[31]<br>*Trautwein, M*<br>*2020*<br>*USA*	Retrospective cohort study<br><br>Academic medical center<br><br>7972 discharges<br><br>C: No<br>P: No	Examination if 30-day readmission prediction instrument has improved performance compared to universal tools<br><br>RR: 12.6%	Model variables (C-statistics: 0.67) (only medication-related)<br>≥ 11 prescription medications on admission (readmission rate: 17%, OR: 1.34 (1.108-1.607), p=0.002
[32]<br>*Criddle, DT*<br>*2021*<br>*Australia*	Retrospective observational study<br><br>Teaching hospital<br><br>1201 discharged patients<br><br>C: No<br>P: No	Improve the predictive performance of a simple risk tool, using modelling with multivariable logistic regression to develop an algorithm aimed at predicting risk of readmission (30, 60, and 90-day readmission was analyzed)<br><br>RR: 25.9% (191)	Model-variables (C-statistics: 0.69) (only medication-related)<br>Polypharmacy (≥ 5 medications), high-risk medications (anticoagulants [B01AA, B01AB, B01AF, B01AX], insulin [A10A], codeine and opioids [R05DA04, N02A], benzodiazepines and z-drugs in patients ≥65 years [N05CF, N05BA], NSAIDs in patients ≥65 years [M01A], antipsychotics [N05A], therapeutic drug monitoring drugs (amiodarone [C01BD01], carbamazepine [N03AF01], clonazepam [N03AE01], ciclosporin [L04AD01], digoxin [C01AA05], everolimus [L04AA18], lamotrigine [N03AX09], lithium [N05AN01], perhexiline [C08EX02], phenobarbital [N03AA02], phenytoin [N03AB02], sirolimus [L04AA10], tacrolimus [L04AD02], theophylline [R03DA04]), diuretics [C03] in patients ≥65 years, chemotherapeutic agents [L01]

[33]<br>*Picker, D*<br>*2015*<br>*USA*	Retrospective cohort study<br><br>Academic medical center<br><br>5507 discharged patients<br><br>C: No<br>P: No	Study the influence of number of discharge medications on the prevalence of 30-day readmission<br><br>20.8% (1147)	RF: Number of discharge medications >6 (OR 1.26, 1.17-1.36; p=0.003)<br>AUCROC = 0.661 (with other non-medication related RFs)
[34]<br>*SanFilippo, S*<br>*2021*<br>*USA*	Single-center retrospective cohort study<br><br>Community teaching hospital<br><br>1386 discharges of patients ≥65 years and ≥5 discharge medications<br><br>C: No<br>P: No	Quantify the total medication risk burden in elderly patients at discharge from the acute care setting and appraise the association of this MedWise Risk Score with 30-day readmissions<br><br>30-day readmission and association with MedWise Risk Score<br><br>RR: MedWise Risk Score ≥20: 19%<br>MedWise Risk Score <20: 11%<br>(p=0.009)	MedWiseRisk Score ≥20 in patients discharged home: OR 1.81, p=0.042<br>Score variables:<br>• Risk of ADE (according to FDA adverse event reporting system, FAERS)<br>• Anticholinergic cognitive burden<br>• Sedative Burden<br>• QTc prolongation risk<br>• CYP450 drug interaction burden
[35]<br>*Leffler, ME*<br>*2019*<br>*USA*	Retrospective cohort study<br><br>Tertiary community academic medical center<br><br>1020 readmissions of patients ≥65 years<br><br>C: No<br>P: No	Determine if adding specific medication regimen parameters to a risk assessment tool increase its ability to predict patients' 30-day readmission risk.<br>Determine if the number of admission and /or discharge medications increased the risk for 30-day readmissions.<br>Determine if specific medication classes (oral corticosteroids, anticholinergic medications, benzodiazepines, non-benzodiazepines hypnotics, opioids, anticonvulsants, anticoagulants, insulin, or sulfonylurea medications; based on Beers criteria and previous completed studies) were associated with 30-day readmission<br><br>RR: NA	Model-variables<br>RF: Increased number or prescribed medications (>12 → greatest increase in predictive value)<br>DG: oral corticosteroids (scheduled and as needed basis), opioids (scheduled and as needed basis), anticonvulsants (scheduled and as needed basis), insulin (scheduled and as needed basis), anticholinergic (scheduled or as needed basis).If multivariate analysis was completed (controlled for patient gender, length of stay, and risk assessment score, opioids and insulin (scheduled) and anticholinergic medications and insulin (as needed or scheduled) significantly predicted 30-day readmission.<br>The predictive value of the tool improved when incorporating medication information, however not enough to consider the tool as a good predictor of 30-day readmission. The AUC improved from 0.583 (AUC) to 0.611 (AUC).<br>Other medication related variables: assistance with medication management, polypharmacy (>7 medications)<br>Scoring ≥5 = high risk, 2-4 = moderate risk, >2 = low risk
[36]<br>*Sieck, C*<br>*2019*<br>*USA*	Retrospective quantitative electronic health record study<br><br>Academic hospital<br><br>6849 readmitted patients ≥65 years<br><br>C: No<br>P: No	Determine the validity to predict 30-day readmission of the BOOST risk stratification tool for patients ≥65 years<br><br>RR: NA	BOOST model variables:<br>Problem medications (aOR 1.571 (1.372-1.798, p>0.001)<br>➔ Polypharmacy (≥10 medications)or high risk medications (insulin, anticoagulants, oral hypoglycemic agents, dual antiplatelet therapy, digoxin, or narcotics)<br>C-statistic of whole tool: 0.631

[37]<br>*Nguyen, HL*<br>*2022*<br>*USA*	Propensity matched cohort study<br><br>Academic safety-net hospital<br><br>87'240 hospitalizations screened and 4027 patients per group were included	Determine if a scoring tool (PARADE model) used to allocate resources for admission medication history review can decrease 30-day readmission and identify patients at high risk for adverse drug events.<br>The intervention was a pharmacy-conducted admission medication history review.<br><br>RR:<br>Intervention group: 11%<br>Control group: 15%<br>(p=0.004)	Medications incorporated in PARADE model:<br>Insulin, phenytoin, opioids, clozapine, metolazone, anticoagulants, antihyperglycemics<br>Each medication category was mapped to a list of potential or actual ADEs:<br>• Anticoagulant → Bleeding (ICD10)<br>• Warfarin → Supra/subtherapeutic levels (results/lab)<br>• Phenytoin → supra/subtherapeutic levels (results/lab) → seizure (ICD10)<br>• Metolazone → hypokalemia (results/lab)<br>• Opioids → respiratory depression (ICD10)<br>• Clozapine → neutropenia (results/lab)<br>• Methadone → QTc prolongation (results/lab)
[38]<br>*Allaudeen, N.*<br>*2011*<br>*USA*	Retrospective observational study<br><br>550-bed tertiary care academic medical center<br><br>6805 patients (10'359 consecutive admissions) discharged from the general medicine service<br><br>C: No<br>P: No	Identify risk factors for 30-day readmission<br><br>RR: 17% (1762)	RF: inpatient use of narcotics (OR 1.33, CI 1.16-1.53), inpatient use of corticosteroids (OR 1.24, CI 1.09-1.42) (only independently associated)
[39]<br>*Witherington, EM*<br>*2008*<br>*UK*	Retrospective case-note review<br><br>Teaching hospital<br><br>108 readmitted patients ≥75 years<br><br>C: Yes → Howard<br>P: Yes → Howard	Identification of communication gaps at hospital discharge for patients ≥75 years readmitted within 28 days.<br>Assess the contribution of incomplete discharge information to readmission.<br>Identification of information that should be included in electronic discharge documents.<br><br>RR: 38% (41) were judged medication-related readmissions and thereof 61% (25) preventable.	RF: Incomplete medication discharge information, 92% thereof preventable (most often medication missing in discharge medication list, resulting in non-administration of this medication in the community)<br>MRPs:<br>• Adverse drug effects (ADEs of new drugs occurring after discharge(e.g. NSAIDs related gastrointestinal bleeding), adverse drug effects of altered drug, occurring before discharge neither addressed nor communicated to the GP (e.g. diarrhea, falls, vomiting), adverse effects of drugs which were stopped with no given reason or monitoring arranged, adverse effect due to medication omitted from medication list)<br>• Failure to address compliance problems<br>• Untreated condition from previous admission (i.e. underprescribing)
[40]<br>*Rosen, OZ*<br>*2017*<br>*USA*	Retrospective cohort study<br><br>Tertiary care teaching hospital<br><br>385 patients<br><br>C: No<br>P: No	Test if medication adherence obtained at hospital admission with the MMAS-4 scale can predict 30-day readmission<br><br>RR:<br>Patients with low and intermediate adherence: 20.0%<br>Patients with high adherence: 903%<br>(p=0.005)	RF: low medication adherence (OR 2.54, CI 1.32-4.90, p=0.005)<br>Adding medication adherence to the HOSPITAL score increased C-statistics from 0.65 to 0.70. The HOSPITAL score itself does not have medication-related variables.

[41] Schoonover, H. 2014 USA	[Retrospective chart review]<br><br>Home care agency<br><br>213 patients ≥50 years and admitted to a home care agency upon hospital discharge and had one of the following conditions: peripheral vascular disease, coronary artery disease, congestive heart failure, hypertension, diabetes, hyperlipidemia, major orthopedic condition, and/or COPD<br><br>C: No<br>P: No	Evaluate the impact of MRCI on the potential for an ADE, unplanned 30-day readmission, and 30-day emergency department visits<br><br>RR: NA	<u>RF: </u>Higher MRCI score (≥22) (odds of experiencing readmission was 5.45 times greater (p=0.026)
[42] Logue, E 2016 USA	Retrospective logistic regression<br><br>Private teaching hospital<br><br>958 admissions (from 568 patients) to a family medicine department<br><br>C: No<br>P: No	Identification of available admission data to predict 30-day readmission risk<br><br>RR: 14%	<u>RF: </u>Polypharmacy defined as ≥6 home medications at admission (OR 2.1; CI 1.3-3.7)
[43] Anderson, RE 2016 USA	Prospective observational cohort study<br><br>Tertiary hospital<br><br>381 patients ≥65 years discharged home and having complete information previously admitted to a medicine department.<br><br>C: No<br>P: No	Determine if cognitive dysfunction (measured by the Short Blessed Test (SBT), the executive function component of the Montreal Cognitive Assessment, and the Trail-Making Test Part B (TMT-B)), in particular impaired executive function, is a risk factor for 30-day readmission<br><br>RR: 21.8% (83)	<u>RF: </u>Independent medication management in combination with impaired cognitive dysfunction (readmission odds increased on average 13% with each point decrease in SBT score (p=0.003) and 9% on average with each 0.01 decrease in TMT-B score (p=0.02). If they were taking more than 7 medications and managing medications independently, the odds increased 16% (p=0.01) and 15% (p=0.03), respectively.
[44] Feigenbaum, P 2012	Structured case series<br><br>Data from 18 hospitals, thereof 5 teaching facilities	Determine to which degree 30-day all cause readmissions are potentially preventable and to generate a robust	<u>Factors related to medication management: (28% (70) of potentially preventable readmissions):</u><br>• Medication errors (e.g. wrong medication, wrong dose) during or after hospital stay (n=79)<br>• Inadequate patient and caregiver understanding of medication management (i.e. inadequate teaching) (n=32)

Canada	537 readmissions<br><br>C: No<br>P: Yes → Clinician judgement	understanding of factors leading to potentially preventable readmissions<br><br>RR: 11% (55) very or completely preventable, 36% (195) slightly or moderately preventable	• Patient discharged without medication documentation in transition of care plan (n=24)
[45]<br>Meldon, S<br>2003<br>USA	Prospective cohort study<br><br>2 urban academic emergency departments<br><br>650 patients ≥65 years in discharged from emergency department<br><br>C: No<br>P: No	Evaluate the utility and predictive ability of a six-item triage risk screening tool (TRST)<br><br>RR:<br>30-day readmission: 14.2% (92)<br>30-day ED revisit: 17.7% (115)	Model variables (AUC = 0.72) (only medication-related):<br>• Five or more medications
[46]<br>Yam, CH<br>2010<br>China	Retrospective analysis<br><br>Health care system (provides about 90% of hospital based health care in Hong Kong)<br><br>603 readmissions (randomly selected)<br><br>C: Yes<br>P: Yes → Clinicians judgement with a developed (unpublished) checklist, based on literature	Identify the magnitude, contributing factors, and costs of avoidable 30-day readmissions<br><br>RR: 16.9%<br>Overall avoidable readmissions: 6.9%<br>40.8% (246) deemed avoidable readmissions of the 603 readmissions analyzed	Medication-related causes of readmissions: side effects of drugs /drug-drug interaction (6.1%), non-compliance with medication or diet (3.0%). Both deemed mostly avoidable (70.4% and 80%, respectively)<br>Discharge with a missing/erroneous diagnosis/therapy (100% avoidable)<br>No significant medication-related risk factors
[47]<br>Blanc, AL<br>2019<br>Switzerland	Retrospective, observational, two-center study<br><br>10'374 Patients admitted to the general internal medicine wards of two hospitals<br><br>1 university hospital and 1 regional hospital<br><br>C: No<br>P: Yes → SQLape algorithm	Derive and validate a predictive model of potentially avoidable 30-day readmissions that includes medication profiles and the identification of specific characteristics of patients readmitted to the general internal medicine wards of two Swiss hospitals<br><br>RR: 7.5% (781) potentially avoidable readmissions	Model variables (c-statistics 0.699)<br>Opioid drug prescription (OR 1.3, CI 1.1-1.6) (only one statistically significant in multivariate analysis)
[48]<br>Dorajoo, SR<br>2017<br>Singapore	Retrospective case-control and retrospective cohort study (2-p study)	Derive and validate a prediction model for 15-day readmission<br><br>RR: NA	Model variables (c-statistics 0.65 and 0.64 (temporal and geographical validation cohort) (medication-related variables):<br>Number of discharge medications (OR 1.06, CI 1.01 – 1.12, p=0.026)

	Tertiary hospital<br><br>1291 patients in three cohorts (derivation (for model derivation, 670 patients, 301 readmitted, 369 controls), temporal (101 patients), and geographical cohort (520 patients) (validation of the derived model))<br><br>C: No<br>P: No		
[49] Frankl, SE 1991 USA	Prospective study of readmissions<br><br>720-bed teaching hospital<br><br>2626 admissions to a medicine department<br><br>C: Yes → Clinician judgment<br>P: Yes → Clinician judgment	Measure the rate of emergent 30-day hospital readmission to analyze the clinical causes and their preventability<br><br>RR:<br>12.5% (327) thereof 8.6% (28) deemed potentially preventable<br>20% (65 cases) of all-cause emergent readmissions were complications of drugs. Of these, 13.84% (9 cases) were preventable. This equals to 32% of all preventable emergent readmissions and to 2% of all emergent readmission.	DRP: Side effects were important in 40 (62%) of patients and toxic drug levels in 13 (20%). The remainder were due to a combination of immunologic reactions, drug-disease interaction, and drug-drug interactions.<br>Adherence: Additionally, 4% (13 cases) of all cause emergent readmissions were poor compliance with medications, of whom 23% were potentially preventable.<br>DG: Chemotherapy [L01] (fever and neutropenia, 16 readmission), trimethoprim-sulfamethoxazole [J01EE01] (10 readmissions, 3 with rash), antiarrhythmics [C01B] (6 readmissions), Steroids/immunosuppressives (1 preventable, over-rapidly tampering of dexamethasone) [H01/L04] (6 readmissions), narcotic analgesics [N02A, opioids] (5 readmissions), digoxin (2 preventable, incorrect dosing) [C01AA05], warfarin (2 preventable [illegible] bleeding) [B01AA03], oral insulins [A10], other antibiotics [J01] (all 3 readmissions), calcium channel blocker [C08], pentamidine (2 preventable, toxicity) [P01CX01], theophylline [R03DA04] (all 2 readmissions), diuretic [C03], sodium chloride (mistakenly continued intake) [A12CA01], acetyl salicylic acid (preventable, contraindication as known gastritis) [B01AC06], tranquillizer [N05C] (all 1 readmission)
[50] McAuliffe, LH 2018 USA	Retrospective cohort study<br><br>719-bed academic medical center<br><br>690 patients who were hospitalized in one of all services and was seen by an inpatient TOC pharmacist<br><br>C: No<br>P: Yes → Clinician judgement	Develop and validate a practical prediction tool to identify patients at highest risk for 30-day readmissions at the time of discharge. The tool should be used by pharmacists to discriminate patients likely to benefit most from continued TOC service<br><br>RR: 14.8% (potentially avoidable)	Model variables of MEDCOINS (c-statistics 0.65) (only medication-related variables):<br>Medication count → 10 or more medications

RF: risk factor; ADE: adverse drug events; ADR: adverse drug reactions; LOS: length of stay; MRR: medication related readmission; MRP: medication related problem; OR: odds ratio; NSAID: non-steroidal anti-inflammatory drug; PIM: potentially inappropriate medication; ED: emergency department; HR: hazard ratio; MRCI: medication regimen complexity index; ICD: international classification of disease

[1] Uitvlugt EB, Janssen MJA, Siegert CEH, et al. Medication-Related Hospital Readmissions Within 30 Days of Discharge: Prevalence, Preventability, Type of Medication Errors and Risk Factors. *Frontiers in pharmacology*. 2021;12:567424. doi:https://dx.doi.org/10.3389/fphar.2021.567424
[2] Cooper JB, Jeter E, Sessoms CJ. Rates and Types of Medication-Related Problems in Patients Rehospitalized Within 30 Days of Discharge From a Community Hospital. *Journal of Pharmacy Technology*. 2020;36(2):47-53. doi:http://dx.doi.org/10.1177/8755122519883642
[3] Whitaker AS, Cottrell WN. What proportion of unplanned re-presentations to an emergency department are medication related and preventable? *Journal of Pharmacy Practice & Research*. 2019;49(6):546-556. doi:10.1002/jppr.1581
[4] Dalleur O, Beeler PE, Schnipper JL, Donze J. 30-Day Potentially Avoidable Readmissions Due to Adverse Drug Events. *J Patient Saf*. Aug 1 2021;17(5):e379-e386. doi:10.1097/PTS.0000000000000346
[5] Dreyer R, Viljoen AJ. Evaluation of factors and patterns influencing the 30-day readmission rate at a tertiary-level hospital in a resource-constrained setting in Cape Town, South Africa. *S Afr Med J*. Feb 26 2019;109(3):164-168. doi:10.7196/SAMJ.2019.v109i3.13367
[6] Toh MR, Teo V, Kwan YH, Raaj S, Tan SY, Tan JZ. Association between number of doses per day, number of medications and patient's non-compliance, and frequency of readmissions in a multi-ethnic Asian population. *Prev Med Rep*. 2014;1:43-7. doi:10.1016/j.pmedr.2014.10.001
[7] Willson MN, Greer CL, Weeks DL. Medication regimen complexity and hospital readmission for an adverse drug event. *Ann Pharmacother*. Jan 2014;48(1):26-32. doi:10.1177/1060028013510898
[8] Banholzer S, Dunkelmann L, Haschke M, et al. Retrospective analysis of adverse drug reactions leading to short-term emergency hospital readmission. *Swiss medical weekly*. 2021;151:w20400. doi:https://dx.doi.org/10.4414/smw.2021.20400
[9] Nikolaus T, Specht-Leible N, Kruse W, Oster P, Schlierf G. [The early rehospitalization of elderly patients. Causes and prevention]. *Dtsch Med Wochenschr*. Mar 13 1992;117(11):403-7. Frühe Rehospitalisierung hochbetagter Patienten. Ursachen und Prävention. doi:10.1055/s-2008-1062325
[10] Ekerstad N, Bylin K, Karlson BW. Early rehospitalizations of frail elderly patients - the role of medications: a clinical, prospective, observational trial. *Drug, healthcare and patient safety*. 2017;9:77-88. doi:https://dx.doi.org/10.2147/DHPS.S139237
[11] Rothwell M, Jukka C, Lum E, Mitchell C, Kyriakides P. Retrospective analysis of emergency readmissions to rural and regional hospitals. *Journal of Pharmacy Practice and Research*. 2011;41(4):290-294. doi:http://dx.doi.org/10.1002/j.2055-2335.2011.tb00106.x
[12] Weir DL, Lee TC, McDonald EG, et al. Both New and Chronic Potentially Inappropriate Medications Continued at Hospital Discharge Are Associated With Increased Risk of Adverse Events. https://doi.org/10.1111/jgs.16413. *Journal of the American Geriatrics Society*. 2020/06/01 2020;68(6):1184-1192. doi:https://doi.org/10.1111/jgs.16413
[13] Glans M, Kragh Ekstam A, Jakobsson U, Bondesson A, Midlov P. Medication-related hospital readmissions within 30 days of discharge-A retrospective study of risk factors in older adults. *PLoS One*. 2021;16(6):e0253024. doi:10.1371/journal.pone.0253024
[14] Barnett NL, Dave K, Athwal D, Parmar P, Kaher S, Ward C. Impact of an integrated medicines management service on preventable medicines-related readmission to hospital: a descriptive study. *Eur J Hosp Pharm*. Nov 2017;24(6):327-331. doi:10.1136/ejhpharm-2016-000984
[15] Koekkoek D, Bayley KB, Brown A, Rustvold DL. Hospitalists assess the causes of early hospital readmissions. *J Hosp Med*. Sep 2011;6(7):383-8. doi:10.1002/jhm.909
[16] Classen DC, Jaser L, Budnitz DS. Adverse drug events among hospitalized Medicare patients: epidemiology and national estimates from a new approach to surveillance. *Jt Comm J Qual Patient Saf*. Jan 2010;36(1):12-21. doi:10.1016/s1553-7250(10)36003-x
[17] Porter M, Quillen D, Fe Agana D, et al. Are Patients Frequently Readmitted to the Hospital Different from the Other Admitted Patients? *J Am Board Fam Med*. Jan-Feb 2019;32(1):58-64. doi:10.3122/jabfm.2019.01.180052
[18] Wetherell M, Sweeney M, Weingart SN. Can Clinicians Predict Readmissions? A Prospective Cohort Study. *J Healthc Qual*. Nov/Dec 2017;39(6):345-353. doi:10.1097/jhq.0000000000000056
[19] Balla U, Malnick S, Schattner A. Early readmissions to the department of medicine as a screening tool for monitoring quality of care problems. *Medicine (Baltimore)*. Sep 2008;87(5):294-300. doi:10.1097/MD.0b013e3181886f93
[20] Aljishi M, Parekh K. Risk factors for general medicine readmissions and association with mortality. *N Z Med J*. May 23 2014;127(1394):42-50.

[21] Schwab C, Korb-Savoldelli V, Escudie JB, et al. Iatrogenic risk factors associated with hospital readmission of elderly patients: A matched case-control study using a clinical data warehouse. *J Clin Pharm Ther*. Jun 2018;43(3):393-400. doi:10.1111/jcpt.12670
[22] Beckner A, Liberty KR, Cohn T. Medication Adherence among Home Health Patients Facing Hospital Readmissions. *MEDSURG Nursing*. 2021;30(6):396-402.
[23] Lau MHM, Tenney JW. Evaluation of Drug-Disease Interactions and Their Association with Unplanned Hospital Readmission Utilizing STOPP Version 2 Criteria. *Geriatrics (Basel)*. Nov 8 2017;2(4)doi:10.3390/geriatrics2040033
[24] Pavon JM, Zhao Y, McConnell E, Hastings SN. Identifying risk of readmission in hospitalized elderly adults through inpatient medication exposure. *J Am Geriatr Soc*. Jun 2014;62(6):1116-21. doi:10.1111/jgs.12829
[25] Ibarra Mira ML, Caro-Teller JM, Rodríguez Quesada PP, Garcia-Muñoz C, Añino Alba A, Ferrari Piquero JM. Impact of a Pharmaceutical Care Program at Discharge on Patients at High Risk of Readmission According to the Hospital Score. *J Pharm Technol*. Dec 2021;37(6):310-315. doi:10.1177/87551225211047607
[26] Pereira F, Verloo H, Zhivko T, et al. Risk of 30-day hospital readmission associated with medical conditions and drug regimens of polymedicated, older inpatients discharged home: a registry-based cohort study. *BMJ Open*. 2021;11(7):e052755. doi:10.1136/bmjopen-2021-052755
[27] Basnet S, Zhang M, Lesser M, et al. Thirty-day hospital readmission rate amongst older adults correlates with an increased number of medications, but not with Beers medications. *Geriatr Gerontol Int*. Oct 2018;18(10):1513-1518. doi:10.1111/ggi.13518
[28] Lohman MC, Cotton BP, Zagaria AB, et al. Hospitalization Risk and Potentially Inappropriate Medications among Medicare Home Health Nursing Patients. *J Gen Intern Med*. Dec 2017;32(12):1301-1308. doi:10.1007/s11606-017-4157-0
[29] Sorensen A, Grotts JF, Tseng CH, et al. A Collaboration Among Primary Care-Based Clinical Pharmacists and Community-Based Health Coaches. *J Am Geriatr Soc*. Jan 2021;69(1):68-76. doi:10.1111/jgs.16839
[30] Fung L, Huynh T, Brush T, Medders K, El-Kareh R, Daniels CE. A Correlation of a Medication-Focused Risk Score to Medication Errors at Discharge. *J Clin Pharmacol*. Nov 2020;60(11):1416-1423. doi:10.1002/jcph.1642
[31] Trautwein M, Schwartz S, Price LL, et al. Developing a Real-Time Prediction Model for Medicine Service 30-Day Readmissions. *Journal of Clinical Outcomes Management*. 2020;27(1):33-40.
[32] Criddle DT, Devine B, Murray K, et al. Developing PHarmacie-R: A bedside risk prediction tool with a medicines management focus to identify risk of hospital readmission. *Research in social & administrative pharmacy : RSAP*. 2021;doi:https://dx.doi.org/10.1016/j.sapharm.2021.08.014
[33] Picker D, Heard K, Bailey TC, Martin NR, LaRossa GN, Kollef MH. The number of discharge medications predicts thirty-day hospital readmission: a cohort study. *BMC Health Serv Res*. Jul 23 2015;15:282. doi:10.1186/s12913-015-0950-9
[34] SanFilippo S, Michaud V, Wei J, Bikmetov R, Turgeon J, Brunetti L. Classification and Assessment of Medication Risk in the Elderly (CARE): Use of a Medication Risk Score to Inform Patients' Readmission Likelihood after Hospital Discharge. *J Clin Med*. Aug 31 2021;10(17)doi:10.3390/jcm10173947
[35] Leffler ME, Elliott DP, Thompson S, Dean LS. Medication-related readmission risk assessment in older adult patients. *Journal of the American College of Clinical Pharmacy*. 2019;2(6):652-659. doi:10.1002/jac5.1104
[36] Sieck C, Adams W, Burkhart L. Validation of the BOOST Risk Stratification Tool as a Predictor of Unplanned 30-Day Readmission in Elderly Patients. *Qual Manag Health Care*. Apr/Jun 2019;28(2):96-102. doi:10.1097/qmh.0000000000000206
[37] Nguyen HL, Alvarez KS, Manz B, et al. Real-Time Risk Tool for Pharmacy Interventions. *Hosp Pharm*. Feb 2022;57(1):52-60. doi:10.1177/0018578720973884
[38] Allaudeen N, Vidyarthi A, Maselli J, Auerbach A. Redefining readmission risk factors for general medicine patients. *J Hosp Med*. Feb 2011;6(2):54-60. doi:10.1002/jhm.805
[39] Witherington EM, Pirzada OM, Avery AJ. Communication gaps and readmissions to hospital for patients aged 75 years and older: observational study. *Qual Saf Health Care*. Feb 2008;17(1):71-5. doi:10.1136/qshc.2006.020842
[40] Rosen OZ, Fridman R, Rosen BT, Shane R, Pevnick JM. Medication adherence as a predictor of 30-day hospital readmissions. *Patient Prefer Adherence*. 2017;11:801-810. doi:10.2147/ppa.S125672

[41] Schoonover H, Corbett CF, Weeks DL, Willson MN, Setter SM. Predicting potential postdischarge adverse drug events and 30-day unplanned hospital readmissions from medication regimen complexity. *J Patient Saf*. Dec 2014;10(4):186-91. doi:10.1097/pts.0000000000000067
[42] Logue E, Smucker W, Regan C. Admission Data Predict High Hospital Readmission Risk. *J Am Board Fam Med*. Jan-Feb 2016;29(1):50-9. doi:10.3122/jabfm.2016.01.150127
[43] Anderson RE, Birge SJ. Cognitive Dysfunction, Medication Management, and the Risk of Readmission in Hospital Inpatients. *J Am Geriatr Soc*. Jul 2016;64(7):1464-8. doi:10.1111/jgs.14200
[44] Feigenbaum P, Neuwirth E, Trowbridge L, et al. Factors contributing to all-cause 30-day readmissions: a structured case series across 18 hospitals. *Med Care*. Jul 2012;50(7):599-605. doi:10.1097/MLR.0b013e318249ce72
[45] Meldon SW, Mion LC, Palmer RM, et al. A brief risk-stratification tool to predict repeat emergency department visits and hospitalizations in older patients discharged from the emergency department. *Acad Emerg Med*. Mar 2003;10(3):224-32. doi:10.1111/j.1553-2712.2003.tb01996.x
[46] Yam CH, Wong EL, Chan FW, et al. Avoidable readmission in Hong Kong--system, clinician, patient or social factor? *BMC Health Serv Res*. Nov 17 2010;10:311. doi:10.1186/1472-6963-10-311
[47] Blanc AL, Fumeaux T, Stirnemann J, et al. Development of a predictive score for potentially avoidable hospital readmissions for general internal medicine patients. *PLoS One*. 2019;14(7):e0219348. doi:10.1371/journal.pone.0219348
[48] Dorajoo SR, See V, Chan CT, et al. Identifying Potentially Avoidable Readmissions: A Medication-Based 15-Day Readmission Risk Stratification Algorithm. *Pharmacotherapy*. Mar 2017;37(3):268-277. doi:10.1002/phar.1896
[49] Frankl SE, Breeling JL, Goldman L. Preventability of emergent hospital readmission. *Am J Med*. Jun 1991;90(6):667-74.
[50] McAuliffe LH, Zullo AR, Dapaah-Afriyie R, Berard-Collins C. Development and validation of a transitions-of-care pharmacist tool to predict potentially avoidable 30-day readmissions. *Am J Health Syst Pharm*. Feb 1 2018;75(3):111-119. doi:10.2146/ajhp170184
